# Magnetic Tensegrity-Enabled Robotic Gripper with Adaptive Energy Barrier for UAV Perching

**DOI:** 10.34133/cbsystems.0535

**Published:** 2026-03-09

**Authors:** Lulu Han, Hao Yang, Luobin Wang, Yuquan Zheng, Jingrui Yang, Yuxuan Fu, Jieliang Zhao, Zhong Wan, Zhigang Wu, Jie Zhang, Jianing Wu

**Affiliations:** ^1^ School of Aeronautics and Astronautics, Sun Yat-Sen University, Shenzhen 518107, China.; ^2^ Department of Chemical and Biological Engineering, The Hong Kong University of Science and Technology, Hong Kong 999077, China.; ^3^ School of Advanced Manufacturing, Sun Yat-Sen University, Shenzhen 518107, China.; ^4^ School of Mechanical Engineering, Beijing Institute of Technology, Beijing 100081, China.; ^5^ Department of Medical Microbiology, Radboud University Medical Center, Nijmegen 6525, Netherlands.; ^6^ School of Mechanics and Aerospace Engineering, Dalian University of Technology, Dalian 116024, China.

## Abstract

Equipping unmanned aerial vehicles (UAVs) with bistable robotic grippers allows them to perch on natural and artificial structures, extending mission duration by minimizing energy consumption during stationary operations. However, achieving both compliant triggering and powerful grasping remains a important challenge, particularly in the absence of active actuators. In this work, we present a magnetic tensegrity-enabled robotic gripper (MTRG) with an adaptive energy barrier by leveraging nonlinear interaction forces between magnets. This physical intelligence enables our MTRG to merge both sensitivity and strength, showcasing a failure-to-triggering force ratio exceeding 2 orders of magnitude, which allows for customized responses to varying interaction requirements. This capability involves gentle triggering and robust grasping, analogous to the behavior exhibited by bats. To enable repeated operation, an integrated inflatable airbag is used to reset the bistable system, allowing for multiple grasping behaviors without manual intervention. When integrated into UAVs, MTRGs showcase reliable perching abilities across diverse scenarios, highlighting the potential of passive mechanisms for enhancing the adaptability of energy barriers to achieve long-duration and high-altitude operations.

## Introduction

Unmanned aerial vehicles (UAVs) are widely employed for missions that demand environmental monitoring and data acquisition across diverse applications, for example, ecological assessment and disaster response, where long-duration aerial presence is essential [1–4]. Maintaining flight requires continuous lift generation, causing energy efficiency to become a critical constraint, especially for lightweight UAV platforms that merely rely on limited onboard battery capacity [5–7]. Recently, significant efforts have been devoted to optimizing control strategies and airframe configurations to improve the energy efficiency of UAVs [8–10]. Despite these advancements, the most effective strategy for reducing energy consumption lies in eliminating the need for continuous lift generation during stationary phases of a mission, i.e., enabling UAVs to temporarily transition into a perched state [11–13].

Perching requires attaching and detaching from environmental settings, either natural (e.g., tree branches) or artificial (i.e., street lamps) ones [14–16]. Early strategies were inspired by the morphological characteristics of birds, incorporating robotic grippers mounted on the ventral side of UAVs to replicate biological perching abilities. These robotic grippers mimic the biomechanical function of avian limbs, enabling UAVs to perform stable and repeatable landing maneuvers on various structures [17]. Among these various strategies, perching mechanisms—typically composed of a digital flexor system for grasping and a tendon locking mechanism for maintaining—have been prominent [18]. This bioinspired strategy enables autonomous initiation of perching and ensures reliable attachment to structures without human intervention [19]. However, effective perching requires highly precise coordination between the UAV’s flight dynamics and the actuation timing of robotic grippers [20–22]. Minor deviations in flight trajectory, orientation, or actuation delay will result in failed attachment attempts or mechanical damage [5,23]. Moreover, current perching mechanisms necessitate either continuous or intermittent energy input to maintain a secure grasp after perching is completed. This ongoing power requirement undermines the intended energy savings of perching and constrains mission duration, especially for UAVs with limited onboard energy reserves [24].

Recent advancements in aerial robotics have highlighted the potential of perching mechanisms that leverage physical intelligence—that is embedding adaptive responses into mechanical structures without relying on external computation [25]. Among these, bistable mechanisms represent a promising pathway to enhance perching efficiency and robustness [26–28]. By integrating bistability into robotic grippers, it becomes possible to achieve passive and autonomous engagement with environmental structures upon contact. Specifically, such robotic grippers can transition between stable states through a passive triggering process, eliminating the need for precise positioning and real-time feedback [29–31]. This markedly reduces control complexity and energy consumption while increasing reliability under conditions of uncertainties, such as dynamic wind disturbances and irregular landing geometries [25]. Moreover, the intrinsic mechanical stability provided by the bistable structures ensures secure attachment during perching, which is crucial for long-duration tasks. These properties collectively position bistable robotic grippers as a compelling solution for advancing the autonomy and resilience of UAV perching systems in complex real-world environments [32].

Despite remarkable progress in the development of bistable structures for robotic grippers, common paradigms are restricted by specialized mechanical configurations, which determine their energy landscape [33]. Specifically, the energy barrier separating the bistable states remains constant once fabricated, limiting the system’s adaptability and hindering performance optimization post-deployment. This static nature poses a trade-off between triggering compliance (required for sensitive engagement) and grasping stability (essential for secure attachment), thus challenging the development of versatile and responsive robotic grippers for grasping and perching [16]. To address this limitation, one strategy involves integrating thermally responsive materials, such as shape memory alloys (SMAs), which enable modulation of energy barriers through temperature-induced phase transformations [34]. While effective in achieving tunability, the thermal regulation suffers from slow response times due to heating and cooling cycles, which significantly constrain the dynamic performance of the bistable robotic grippers [35]. In response, recent efforts explored the incorporation of active actuators to dynamically modulate energy barriers in real time, achieving a functional balance between compliant triggering and stable grasping [16]. Although such approaches improve responsiveness, they still rely on external control inputs and energy supply, thereby increasing system complexity and reducing energy efficiency. Notably, current strategies for enhancing the adaptability of energy barriers remain predominantly active, lacking mechanisms that allow for passive routes in response to diverse environmental interactions.

**Fig. 1.**
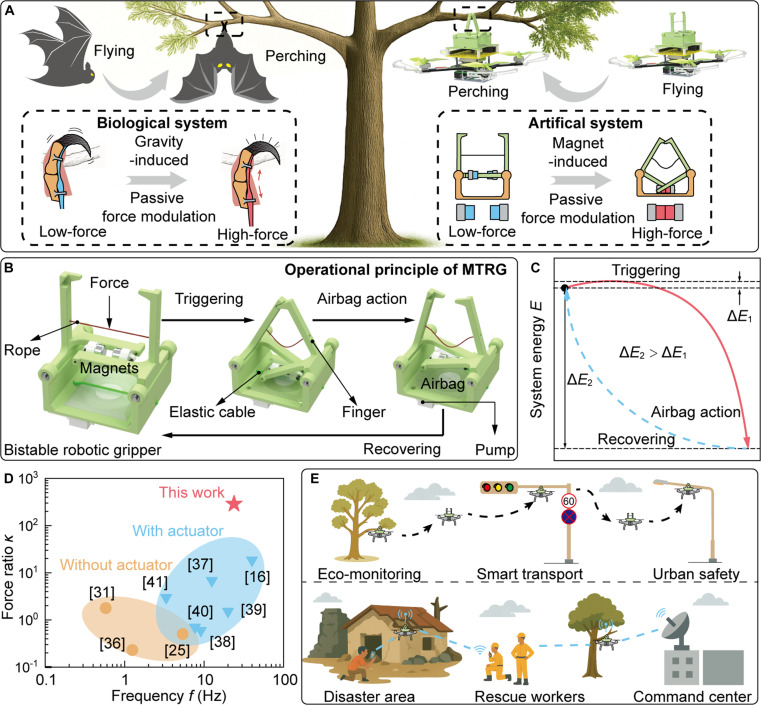
Compliant and powerful UAV perching via MTRG. (A) Perching behavior of biological and artificial systems. (B) Operational principle of our robotic gripper. (C) Energy variation of MTRG during operation. (D) Comparison of switching frequency *f* and force ratio *κ* between our paradigm and current bistable robotic grippers. Here, *κ* is defined as the ratio between failure force and triggering force. (E) Application demonstrations of our robotic gripper that is integrated into UAVs for implementing tasks such as grasping and perching, showing its potential for diverse scenarios.

In nature, bats exhibit remarkable capability to hang stably in an inverted gesture through a passive locking mechanism (Fig. 1A). This mechanism operates such that, once a bat grasps an object, its body weight naturally generates tensions in the legs and feet, which in turn tighten the tendons connected to the toes. This tendon-driven response allows the toes to passively and securely grasp surrounding structures, such as branches, rocks, and ceilings, without requiring any active muscular engagement. Inspired by this biological behavior, we present a magnetic tensegrity-enabled robotic gripper (MTRG) that utilizes bistable mechanics to implement rapid finger closure, which occurs within ~40 ms through a controlled transition between 2 stable states (Fig. 1B). During this transition, the nonlinear amplification of magnetic interaction forces effectively enables the bistable structure to demonstrate adaptive energy barrier, facilitating compliant triggering and powerful grasping, analogous to the capability shown by bats, as shown in Fig. 1C. This passive mechanism significantly enhances the force ratio *κ*, defined as a ratio between failure force to triggering force, thus resolving a fundamental trade-off that has long restricted the performance of the bistable robotic grippers: the balance between compliant triggering and powerful grasping, as illustrated in Fig. 1D and Table S1 [16,25,31,36–41]. This design exemplifies the principles of embodied physical intelligence, wherein mechanical structures themselves contribute to adaptive and functional behaviors without depending on active control or complex computation. Thus, the MTRG provides a robust yet inherently compliant solution for safe, reliable, and efficient grasping across a wide range of applications. To further demonstrate its versatility, the MTRG has been integrated into UAVs, where it enables perching capabilities on diverse substrates, as showcased in Fig. 1E. This integration significantly enhances UAV functionality by supporting long-duration operations and sustained high-altitude tasks without continuous power expenditure. These results underscore the potential of tensegrity-inspired, magnetically actuated systems in advancing next-generation robotic grippers and aerial robotic platforms.

## Materials and Methods

### General design and operational principle

To accomplish passive mechanisms that enable compliant triggering and powerful grasping, analogous to that of bat hindlimb, we present a magnetic tensegrity-enabled paradigm for a bistable robotic gripper, as exhibited in Fig. 1B (Fig. S1 and Movie S1). This configuration draws inspiration from a classical anti-gravity tensegrity structure, which comprises 2 rigid frames connected by 5 tensioned cables [42]. To allow the stable structure to exhibit bistability, the central cable is replaced with pre-stretched springs [25,43]. However, a critical limitation of this spring-based configuration is that the energy barrier between stable states becomes invariable once the structural parameters are determined, preventing a customized trade-off between compliant triggering and powerful grasping. This constraint greatly limits its applicability in scenarios such as UAV perching.

To address this defect, we introduce magnetic interactions as the primary mechanism for maintaining structural stability in the tensegrity. Specifically, our paradigm features 2 finger-like rigid frames connected to a base via rotational hinges. Two high-strength neodymium magnets (diameter: 10 mm, thickness: 10 mm) are mounted on each finger and aligned so that their surfaces remain parallel through hinged attachments. The fingers are connected to the base with stainless steel sliding bearings. Both fingers and the base are structural components fabricated by 3D printing with polylactic acid (PLA). A nonelastic crystal rope is used to maintain the stability of the robotic gripper, forming a balance between magnetic force and tensile force that defines the first stable state (i.e., state 1). When an external force is applied to the rope, this equilibrium is perturbed, allowing the magnetic force to dominate and pulling the magnets together, resulting in the second stable state (i.e., state 2). In this state, the fingers interlace and securely enclose the object. Owing to nonlinear relationships between magnetic force and the gap between the magnets, the transition from states 1 to 2 requires fairly small external input, which enables passive and compliant triggering. Conversely, reversing this transition demands significantly larger force, thus ensuring a firm and stable grasp. This bistable magnetic tensegrity mechanism provides an effective balance between sensitivity and strength, making it highly suitable for adaptive robotic applications. Although the application of asymmetric energy barriers has been mentioned in other studies, this characteristic has not been explored for improving the operational capability of bistable robotic grippers [44].

Releasing a grasped object presents a significant challenge due to powerful magnetic forces that maintain the MTRG in the stable state 2. This robust force hinders passive reversal to the initial configuration, thus necessitating an active mechanism to facilitate state resetting. Therefore, we integrate a pneumatic actuator (i.e., an inflatable airbag) into the base of the MTRG (*m* = 75.23 g). During the release phase, compressed air is introduced into the airbag through a pump. As the airbag inflates, it generates an upward thrust force that acts against the magnets. This force is sufficient to overcome the magnetic attraction, thereby enabling the separation of the magnets and restoring the system to its original, pre-engagement configuration. Subsequently, we apply an elastic cable (marked in green) to ensure a rapidly repeatable recovery process. Once the magnets are disengaged, the elastic cable made of rubber applies a restoring force to gradually deflate the airbag. This hybrid actuation approach, i.e., combining magnetic bistability for passive, compliant, and powerful grasping with pneumatic assistance for active release, offers a robust and energy-efficient solution for repeatable grasping applications. It enables reliable switching between stable states while minimizing the need for continuous power input, making it particularly well-suited for use in mobile systems such as UAVs, where compactness, adaptability, and power efficiency are critical.

### Fabrication of airbag recovering system

The airbag-enabled recovery system is composed of an airbag, an air connector, air pipes, a 3-way pipe, a steel pipe with narrow seams, and an air pump. The airbag is fabricated of 2 pieces of polyethylene (PE), which are sealed by a heat pressing process. The middle part of the airbag uses an air connector component printed with PLA to form an air guide tube, which is connected to one end of a 3-way pipe through soft rubber air pipes. The connection between the air connector and the airbag is achieved by bonding and sealing with instant adhesive to ensure airtightness.

The system applies a miniature diaphragm pump powered by a DC voltage of 3.7 to 5 V. The pump is connected through air pipes and a 3-way connector, and it can offer a pressure difference of ~66 kPa under a 5-V supply. The remaining port of the 3-way connector is linked to a steel pipe, which serves 2 functions. First, the pressure requires only about 28 kPa; excessive pressure could potentially cause damage. Here, the steel pipe allows air leakage through a narrow seam to reduce the pressure acting on the airbag. Second, the steel pipe enables passive deflation of the airbag. Due to the simple single-port design, the airbag is capable of inflating but cannot deflate rapidly when the pump is powered off. The steel pipe provides a pathway for deflation. To accelerate air release, an elastic cable is used to compress the airbag and create an additional pressure gradient.

The on/off switching of the diaphragm pump is controlled via a pulse width modulation (PWM) controller commonly used in model aircraft. By utilizing an auxiliary PWM output channel from the UAV, it is possible to regulate whether 5-V DC is supplied to the pump, thereby enabling direct integration of the pump’s operation into the UAV control system.

### Capturing rapid grasping behavior

A high-speed camera (Phantom VEO-E 310L, USA), fitted with a micro-lens (Canon EF 100 mm f/2.8L IS USM, Japan), is used to record the state transitions of the robotic gripper. To resolve the transient dynamics, the sampling frequency is set to 3,300 fps.

### Measurement of quasi-static mechanical behavior

The quasi-static mechanical response of the triggering process is measured by using a uniaxial tensile testing machine (ESM303, Mark-10, USA). The integrated force sensor has a maximum capacity of 10 N and a resolution of 0.005 N. For each triggering experiment, cylindrical stick is fixed to the testing apparatus and moved vertically toward the robotic gripper’s rope at a speed of 30 mm/min. The displacement begins from a noncontact position and proceeded until the gripper fully executed the triggering action. The resulting force–displacement curves are obtained from the measurements. The measurement of the gripper’s failure force is conducted using a force sensor with a maximum capacity of 50 N and a resolution of 0.02 N.

### Assembly of MTRG-enabled UAVs

The UAV platform with a mass of *m* = 926.48 g consists of a 250-mm diagonal motor-to-motor frame, a Pixhawk 4 flight controller, 4 sets of T-motor F60 V4 brushless motors with BLHELI32 45A electronic speed controllers, a 5-V voltage regulator module, a 4S LiPo battery, a remote-control transceiver, a data transmission antenna, a global position system (GPS) module, an optical flow sensor, and onboard devices. The onboard devices are categorized into 2 types: a communication relay router and a monitoring and sensing system. The communication relay is performed using a Gee1S router. The monitoring and sensing system employs a Raspberry Pi 4B as the sensing server, which collects, records, and transmits data from various sensors, including temperature, humidity, light intensity, and biological sensors, as well as a camera.

### Flight endurance testing method for UAVs

To compare the energy consumption of a UAV during perching and hovering states, voltage and current sensors are used to monitor the UAV’s power supply during task execution. In the perching state, the energy consumption is minimal, with the total system current measured at ~0.4 A (supply voltage of 16.8 V, corresponding to a power of about 6.72 W) applying a laboratory oscilloscope. In contrast, during flight, the current is measured at around 18 A (supply voltage of 16 V, corresponding to a power of ~288 W). Due to the large discrepancy in current magnitude, calculating precise energy consumption by separately measuring voltage and current proved inaccurate, as the cumulative integration error was too high. Since the UAV is powered by a lithium battery, whose energy is closely related to its voltage, the fully charged battery voltage used in the experiment is 16.8 V. Generally, the battery can be considered depleted when its voltage drops to 14.8 V under low-power conditions, or to 14 V under high-power flight conditions. Within this voltage range, the energy consumption can be evaluated by monitoring the battery voltage drop.

### UWB-based station positioning method

To enable accurate positioning in environments where GPS signals are degraded or unavailable, we implement an ultra-wideband (UWB)-based positioning system. Here, 3 perching UAVs are equipped with UWB communication modules and designated as base stations. Utilizing an electromagnetic ranging principle analogous to that of GPS, the system measures the distances between a mobile UAV that equipped with UWB tag modules and each of the base UAVs. These measurements are processed using trilateration algorithms to compute the precise local position of the mobile UAV within the operational area. By using perching techniques to achieve stable and sustained positioning, the base UAVs functioned as quasi-fixed reference nodes in 3-dimensional (3D) space. This strategy establishes a robust spatial reference framework that ensures consistent and accurate localization performance, all while eliminating dependence on external infrastructure.

## Results

### Parameter determination of MTRG

**Fig. 2.**
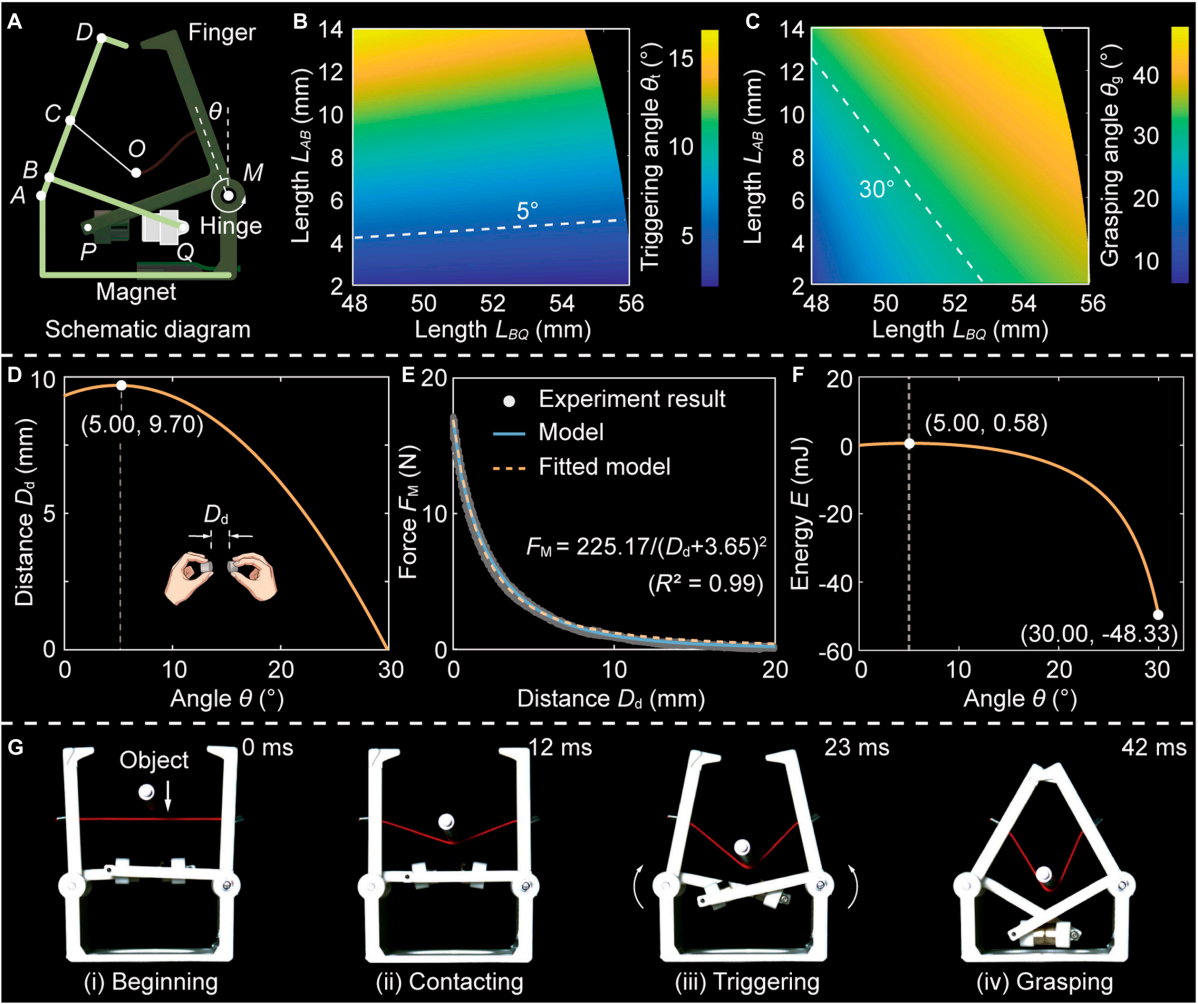
Determination of geometrical parameters for MTRG. (A) Schematic diagram of the robotic gripper. The angle between the finger and the vertical direction is denoted as *θ*, at which the moment exerted by the hinge is marked *M*. The 2 magnets are simplified as points *P* and *Q*. Effects of both structural lengths *L*_*AB*_ and *L*_*BQ*_ on the (B) triggering angle *θ*_t_ and (C) grasping angle *θ*_g_. (D) Relationship between angle *θ* and distance *D*_d_ between the points *P* and *Q*. When the angle reaches 5.00°, the distance *D*_d_ reaches the maximum. (E) Effects of distance *D*_d_ on magnetic force *F*_M_. (F) Robotic energy *E* during stable state transition. (G) Snapshots of our robotic gripper during grasping a free-fall cylindrical object.

To determine structural parameters, the MTRG is simplified as shown in Fig. 2A. Due to the structural symmetry, only one-half is considered in the analysis. In this representation, the endpoints of the finger are labeled as points *A* and *D*, respectively. The connections between the finger and components are identified by points *A*, *B*, and *C*, and the attachment points of the magnets to the structure are marked as points *P* and *Q*. Additionally, the angle between the finger and the vertical axis is defined as *θ*, and the moment generated at the hinge is denoted by *M*. As an external force is applied to the center of the rope (i.e., point *O*), the MTRG undergoes a state transition. Based on the geometric relationships among the structures, a theoretical model is developed to further analyze the behavior of the robotic gripper (Figs. S2 to S4).

The initial phase of our analysis focuses on the triggering process of our MTRG, which plays a critical role in determining sensitivity of the robotic systems. The primary objective is to minimize the triggering angle *θ*_t_ to achieve gentle activation. Based on our model, angle *θ*_t_ is predominantly determined by the geometric configuration of 2 key structural parameters: lengths *L*_*AB*_ and *L*_*BQ*_, respectively. As showcased in Fig. 2B, these structural dimensions demonstrate a well-defined and predictable influence on the angle. In principle, it is possible to design the system such that *θ*_t_ approaches zero, which will result in an extremely sensitive trigger. Nevertheless, this level of sensitivity significantly reduces the resistance to external disturbances, thus increasing the risk of unintentional activation due to mechanical vibrations. Hence, this trade-off between trigger sensitivity and anti-interference robustness must be carefully balanced to ensure optimal performance. This balance can be customized according to the application scenarios. For example, we set the triggering angle *θ*_t_ at 5°, a value identified as an optimal compromise. At this angle, MTRG maintains ease of activation and shows enhanced resistance to inadvertent triggering caused by minor perturbations (Fig. S5 and Movie S2).

Following the analysis of the triggering process, we investigate the configuration after MTRGs grasp an object, corresponding to stable state 2. In this state, it is essential that the fingers reach a closing angle of 30° for our MTRG to perform a secure grasp without causing physical interference between fingers. This angular constraint ensures mechanical integrity and functional alignment during operation. Here, the geometric relationship required to achieve this MTRG is shown in Fig. 2C, which complements the earlier analysis depicted in Fig. 2B. By considering both triggering and grasping requirements, a feasible design region for the geometric parameters can be determined. The final values for lengths *L*_AB_ and *L*_*BQ*_ are selected based on these constraints to ensure MTRGs to perform effectively across operational states. The finalized structural parameters used in the design of the MTRG are summarized in Table S2, which reflects the outcome of this comprehensive analysis.

After establishing the initial structural parameters, we characterize the relationship between the rotation angle *θ* and the distance *D*_d_ between the magnets (Fig. 2D). This analysis reveals a nonmonotonic correlation: When the angle *θ* reaches 5.00°, the distance *D*_d_ attains a maximum value of 9.70 mm. The presence of the nonmonotonic behavior is a critical indicator of the system’s bistable property. Subsequently, we experimentally characterize the magnetic force *F*_M_ as a function of the distance *D*_d_ to gain deeper insights into the underlying mechanics. As presented in Fig. 2E, this relationship is fitted as $F_{M}=225.17/{\left(D_{d}+3.65\right)}^{2}$. This inverse-square dependency underscores the highly nonlinear nature of magnetic forces and serves as the foundation for constructing the system’s energy landscape. The influence of structural parameters on force *F*_M_ is showcased in Fig. S6. Applying the relation $E=\int F_{M}dD_{d}$, we derive the energy *E* associated with the state transitions (Fig. 2F and Fig. S6). The resulting energy landscape highlights a distinct asymmetry between the forward and reverse transitions. Specifically, transitioning from the initial state (state 1) to the grasping state (state 2) requires merely 0.58 J of energy input. This low-energy threshold enables gentle and efficient activation of our MTRG. Conversely, reversing the transition from the grasping state back to the initial state requires a higher energy input of 48.88 J. This stark contrast—an energy requirement nearly 85 times greater—creates a substantial energy barrier, effectively locking the MTRG in the grasping state and ensuring reliable object retention. Grasping behavior is shown in Fig. 2G and Movie S3, which provides sequential snapshots of the MTRG in operation. These images capture the MTRG successfully performing object operation tasks, such as grasping cylindrical objects, with a triggering process of 42 ms, showing the system’s practical effectiveness in real-world applications.

### Compliant triggering of robotic gripper

**Fig. 3.**
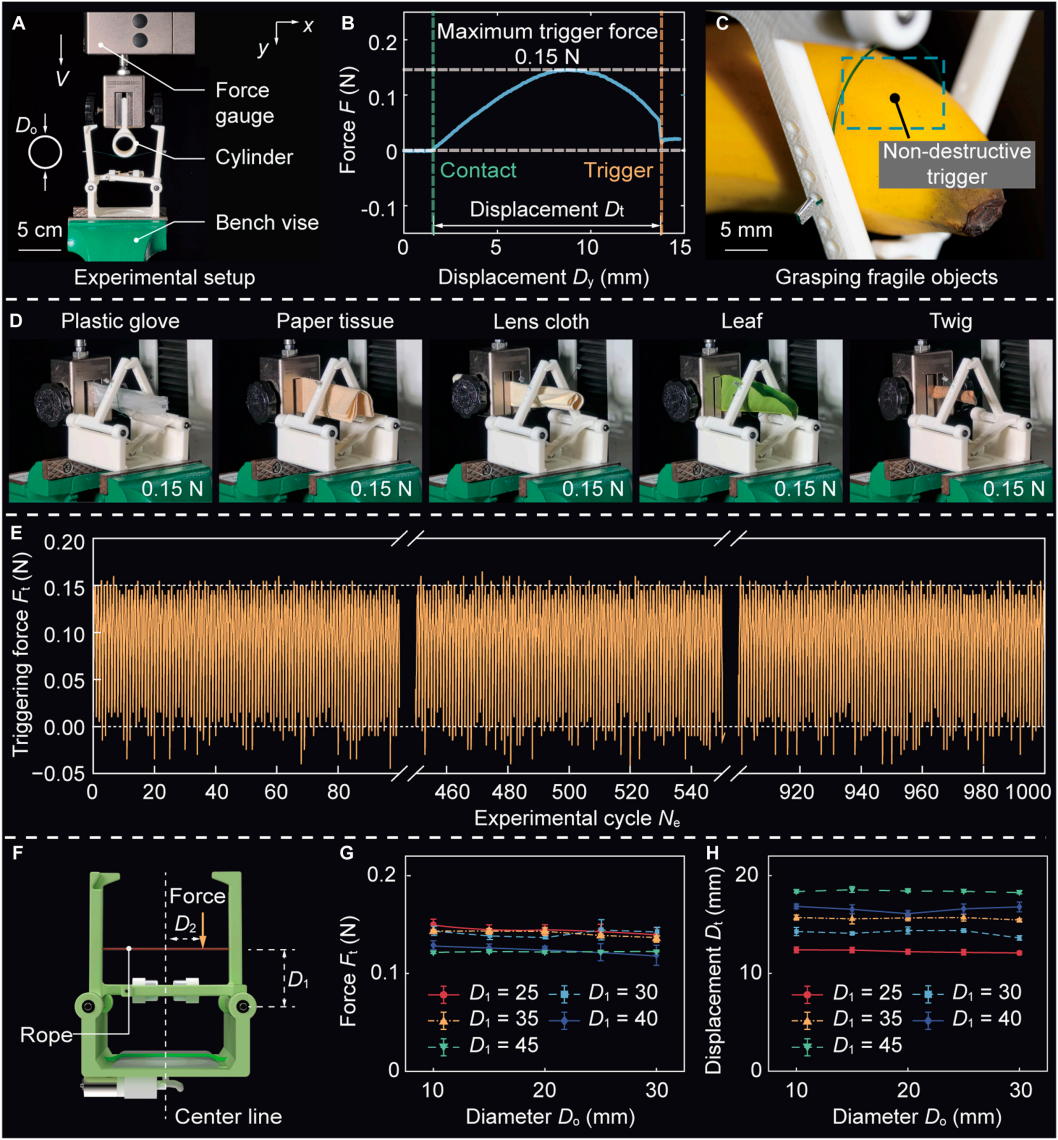
Triggering force evaluation of MTRG. (A) Experimental setup for evaluating the triggering force *F*_t_. The diameter of an object is denoted as *D*_o_. (B) Force–displacement (*F*-*D*_*y*_) curve of our MTRG. (C) Compliant triggering behavior while grasping fragile objects, such as banana. (D) Robotic gripper gently grasping a range of objects made of diverse materials. (E) Characterization of cyclic stability of our MTRG. (F) Position for the placement of the rope and the application of the force. *D*_1_ denotes the longitudinal distance of the mounted position of the rope from the hinge, and *D*_2_ represents the distance from the force application point to the center line. Effects of distance *D*_1_ on (G) triggering force *F*_t_ and (H) triggering displacement *D*_t_.

The low forward energy barrier greatly reduces the required force for state transition, enabling highly compliant triggering. Fig. 3A shows an experimental setup to evaluate the triggering force *F*_t_ of our MTRG when interacting with an object with a diameter of *D* (Movie S4). This setup enables the measurement of the force exerted by the robotic gripper during the state transition from the open to the closed configuration. The force–displacement relationship under quasi-static implementation, captured in Fig. 3B, offers insights into the mechanical behavior of the robotic system throughout the triggering process. The longitudinal displacement of the center of the rope, denoted as point *O* in Fig. 2A, is recorded to be 12.22 mm. During this process, the maximum force *F*_t_ is merely 0.15 N, which highlights the inherently gentle interaction between the MTRG and the object during state transition, a critical factor when handling delicate or fragile objects. This finding underscores the suitability of our MTRG for applications requiring delicate operation. Specifically, the minimal force required to initiate grasp significantly reduces the likelihood of damaging sensitive materials or structures. A notable example of this capability is the ability to securely grasp a soft fruit—such as a banana—without causing visible indentation (Fig. 3C). This further confirms the capacity of MTRG for high compliance and low mechanical intrusion, making it fit for use in environments where object integrity must be preserved. Notably, as the contact speed increases, the triggering force shows a decreasing trend (Fig. S7).

This low-force interaction is shown to be independent of the material properties of the object. As demonstrated in Fig. 3D, the triggering mechanism relies solely on the geometric compression between the rope and the object, rather than on friction or specific material attributes. Therefore, the MTRG maintains consistent performance across a wide range of materials, enhancing its versatility and reliability in different practical applications. Furthermore, a 1,000-cycle triggering experiment is performed to evaluate the long-term durability and operational consistency of our MTRG, with results shown in Fig. 3E (Fig. S8). This experiment is designed to emulate prolonged usage conditions and evaluate the mechanical reliability of the triggering mechanism under repeated actuation. Over the course of 1,000 cycles, the force *F*_t_ remains consistent, maintaining an average value of ~0.15 N with minimal fluctuation, as shown in Fig. S9. This stable force indicates fine mechanical robustness and repeatability of the robotic system. The ability to hold consistent performance over numerous cycles underscores the reliability in high-frequency manipulation. Such repeatability is critical for deployment in industrial automation and repetitive pick-and-place tasks.

To further enhance the compliant triggering behavior of the MTRG, we conduct a detailed analysis of how the positioning of the rope anchoring point and the force application point, denoted as *D*_1_ and *D*_2_, affects the triggering force *F*_t_, as shown in Fig. 3F. Specifically, *D*_1_ represents the longitudinal distance between the anchoring point and the hinge, while *D*_2_ refers to the lateral distance from the point of force application to the central axis of the MTRG. First, experimental results reveal that increasing *D*_1_ leads to a modest reduction in the triggering force *F*_t_, while a notable increase in the triggering displacement *D*_t_ occurs. For instance, when grasping an object with a cross-sectional diameter of *D*_o_ = 10 mm, increasing the anchoring distance from 25 to 45 mm results in merely a 0.03 N reduction in force *F*_t_, with the corresponding displacement *D*_t_ increasing by 3.45 mm, as shown in Fig. 3G and H. This behavior indicates that increasing *D*_1_ not only helps lower the triggering force but also enhances the MTRG’s tolerance to external disturbances by extending the mechanical travel required to complete the grasp. Within the structure, the rope position *D*_1_ can be adjusted via the holes designed on the finger (Fig. S1). Such design flexibility allows for altering its compliance and robustness based on the specific requirements. Then, we observe that the location of the applied force relative to point *O* (i.e., *D*_2_) significantly influences force *F*_t_ (Fig. S10). When the force is applied directly at point *O*, the triggering force is minimized, measured at 0.12 N. As the contact point moves farther from point *O*, the force gradually goes up, reaching a peak value of 0.15 N. At this maximum triggering force, the displacement required to accomplish triggering extends to 18.40 mm. These design principles indicate that tuning either rope placement *D*_1_ or force application *D*_2_ alters both the compliance and responsiveness of the MTRG. In addition, the sensitivity analysis of triggering displacement and maximum energy is conducted, as shown in Fig. S11. This tunability is particularly advantageous in applications where minimizing interaction forces or maximizing stability under external perturbations is critical.

### Powerful grasping of robotic gripper

**Fig. 4.**
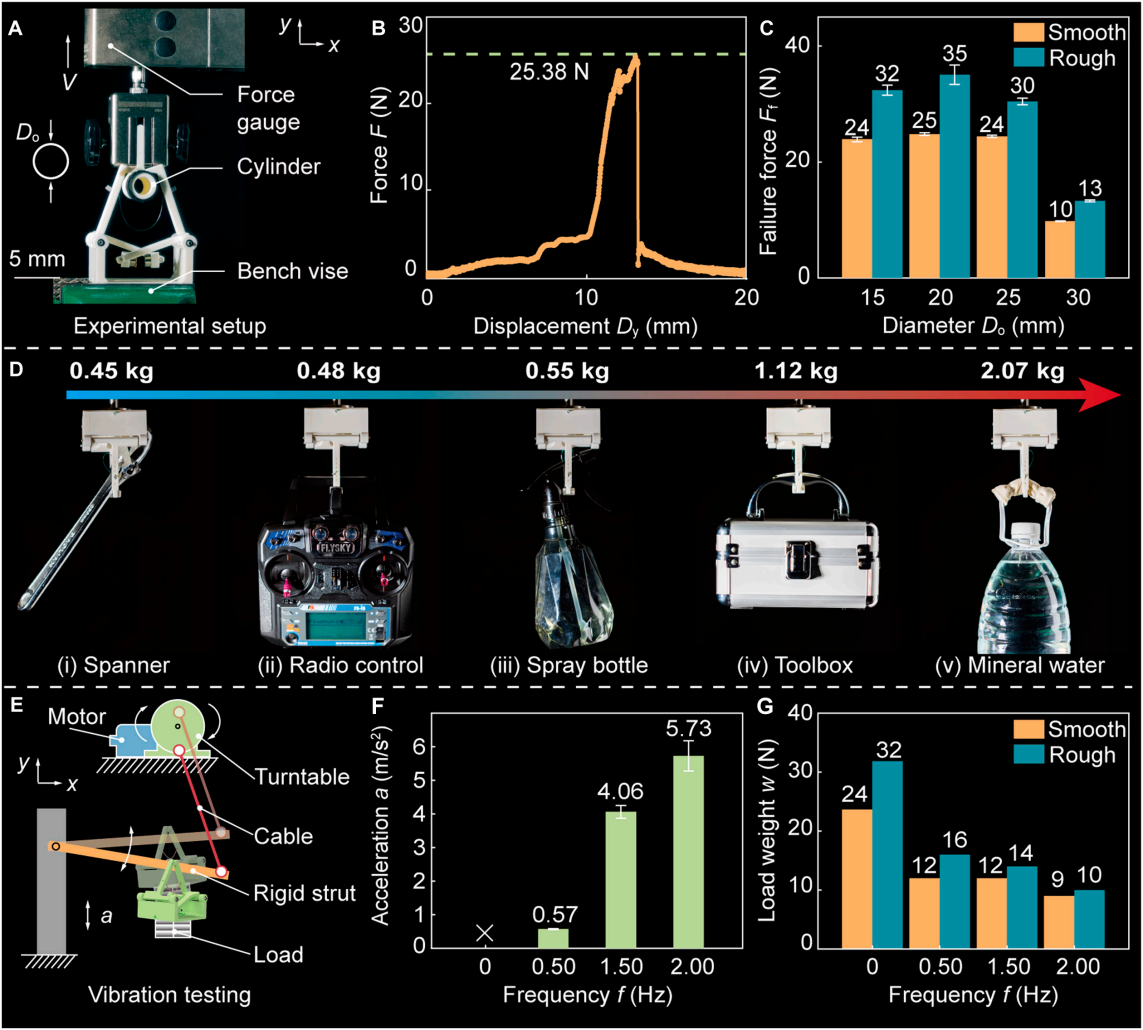
Failure force characterization of MTRG. (A) Experimental setup for evaluating the failure force *F*_f_. (B) Force–displacement (*F*–*D*_*y*_) curve of our MTRG during detaching. (C) Effects of the diameter of the object on force *F*_f_. (D) Our MTRG stably grasping objects with diverse masses, ranging from 0.45 to 2.07 kg. (E) Diagram of experimental setup used to characterize the influence of vibration on grasping stability. (F) Variation of vertical acceleration and (G) load-bearing capacity under diverse vibration frequencies *f*.

After executing the grasping action, we also characterize the mechanical stability of the MTRG by measuring its failure force *F*_f_, which is defined as the critical force at which the object detaches from the robotic gripper, as shown in Fig. 4A (Movie S5). In this experimental setup, the MTRG is rigidly mounted onto a bench vise, while a cylindrical object with a smooth surface (*D*_o_ = 15 mm) is subjected to an axial pulling force employing a testing machine. This experiment simulates the detachment process under an increasing tensile load, capturing the robotic gripper’s passive holding performance. The corresponding force–displacement curve is shown in Fig. 4B. The curve reveals that the gripper achieves a peak failure force of 25.38 N, which shows about a 200-fold increase relative to its triggering force. This dramatic amplification highlights a crucial design advantage: The MTRG can transition seamlessly from a compliant triggering mode to a powerful grasping mode—without the requirement for active force regulation or closed-loop control. This dual-mode functionality overcomes the trade-off between compliance and robustness in robotic systems.

It is important to note that in practical scenarios, the surfaces of grasped objects are often nonsmooth due to surface texture, material irregularities, or functional coatings. To accurately reflect these real-world conditions, we conduct an additional experiment in which a velvet cloth is affixed to the surface of the cylinder, effectively converting it from a smooth to a rough surface. This modification results in a 30% increase in the measured force *F*_f_ (Fig. 4C). The enhanced friction between the MTRG and the object contributes to the improved holding performance, highlighting the adaptability to diverse surface conditions. Nevertheless, the performance showcases size-dependent limitations. For example, when the diameter of the object exceeds 25 mm, the force experiences a sharp decline. This behavior is attributed to the object exceeding the robotic gripper’s effective operational range, thus preventing full finger closure. This geometric constraint illustrates the importance of matching the kinematic envelope to the object size for maximum effectiveness. Despite these size limitations, the high failure force demonstrated across compatible object sizes enables the MTRG to reliably transport objects in varying masses, ranging from 0.45 to 2.07 kg (Fig. 4D). This wide payload capability, achieved without active modulation, makes MTRGs well-suited for applications demanding delicate engagement and stable holding.

While the above experiments are designed to characterize the failure force of the robotic gripper under quasi-static laboratory conditions, it is critical to account for the dynamic environments in which the MTRG is intended to operate. Specifically, when deployed on UAVs for perching tasks, for example, stabilizing on tree branches, the system is subject to external disturbances, most notably wind-induced oscillations. The dynamic factors can significantly alter the effective load-bearing capacity of the MTRG, thereby compromising grasp stability and reliability. To investigate the impact of such environmental perturbations, we perform an experiment to simulate the effect of branch oscillation on the failure force of the MTRG, as showcased in Fig. 4E. In this setup, the UAV-mounted MTRG perches on a long strut (analogous to a tree branch), which is then excited using a cable (marked in red) to induce controlled oscillatory motion at different frequencies *f*. This experimental framework simulates real-world disturbances experienced by UAVs when inhabiting extreme natural environments, such as wind disturbances (Movie S6). When the frequency *f* is held at 0 Hz, representing a static environment, the acceleration *a* experienced by the MTRG remains negligible. Under this condition, the MTRG achieves a maximum load capacity of 32 N, reflecting its intrinsic mechanical robustness in the absence of external dynamic influences. However, as the frequency increases, the inertial effects induced by branch movement become pronounced, as shown in Fig. 4F. At a frequency of 2 Hz, the MTRG undergoes a peak longitudinal acceleration of 12.20 m/s^2^. This elevated dynamic loading imposes significant stress on the gripper–object interface, leading to a substantial reduction in the holding force. Specifically, under this condition, the failure force is attenuated by 68.75% relative to the static baseline (Fig. 4G). To better reflect real-world operation conditions, we evaluate the grasping stability under different disturbances and torsional loads (Fig. S12). Among these results, the MTRG showcases a similar phenomenon of reduced load-bearing capacity. This pronounced degradation highlights the sensitivity of grasp stability to oscillatory disturbances, which are common in aerial manipulation scenarios. These findings underscore the necessity of system design for aerial robotic platforms. The weight of UAVs is reasonably customized according to the environmental conditions of the application to improve the stability of grasping.

### State recovery of MTRGs via an airbag

**Fig. 5.**
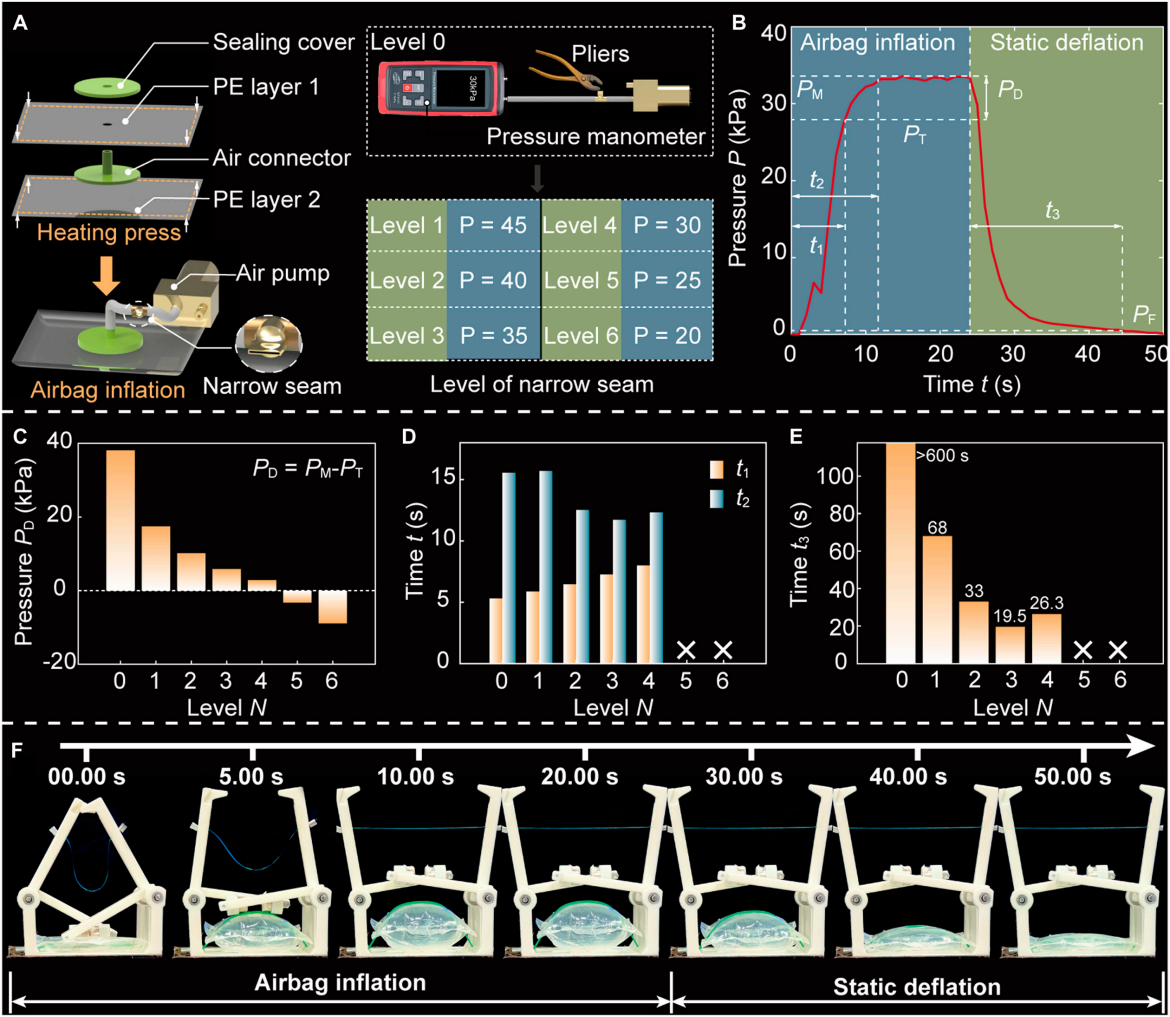
State recovery of our MTRG through an airbag. (A) Fabrication process of an airbag. Six narrow seams are employed to tune pressure, and the corresponding tunable pressures are showcased in the right table. (B) Dynamic pressure response of the airbag during inflation and deflation phases. (C) Pressure differential *P*_D_ between the maximum pressure *P*_M_ and the critical threshold *P*_T_. (D) Effects of seams on 2 temporal parameters *t*_1_ and *t*_2_. (E) Deflation duration of an airbag with diverse seams. (F) Snapshots of airbag inflation and static deflation.

To enhance the reusability of the MTRG, a lightweight airbag (mass: 1.50 g) is integrated at its base, with the fabrication process illustrated in Fig. 5A and Fig. S13. The airbag is formed by thermally bonding 2 layers of PE film to create a sealed chamber, which is actuated via an external air pump. A 3-way pipeline connector is employed to interface the airbag, air pump, and a pressure-regulating valve. To retain a lightweight design, the pressure regulation is achieved by compressing a thin-walled cylindrical valve, generating narrow seams that enable controlled pressure modulation. Due to the minute size of narrow seams, both visual inspection and manual control are challenging. To address this limitation, a pressure manometer is connected to the branch of the 3-way pipeline connector leading to the airbag, enabling precise monitoring during the fabrication of narrow seams with varying pressure regulation capacities. Seven distinct seams are fabricated, labeled as levels 0 to 6, where level 0 represents a fully sealed thin-walled cylindrical tube with no seam present, as demonstrated in Fig. S14. Then, the corresponding tunable air pressures are showcased in the right table of Fig. 5A. By varying the seam size, the internal air pressure of the airbag can be finely tuned within a range of 20 to 45 kPa.

The dynamic pressure response of the airbag during both inflation and deflation phases is shown in Fig. 5B. To quantitatively evaluate the performance of the pressure regulation system, we analyze the critical pressure threshold *P*_T_ required to reset the robotic gripper. Experimental results confirm that the gripper reliably returns to its initial configuration when the airbag pressure reaches *P*_T_ = 28 kPa (Fig. S15). Based on this, 2 temporal parameters are defined to describe the inflation process. The first parameter *t*_1_ represents the time required for the air pressure within the airbag to reach the critical threshold under a given narrow seam. Due to the inherent fluctuations in pressure, the second parameter *t*_2_ is defined as the time taken for the air pressure to reach 98% of its maximum value *P*_M_. To further quantify the effectiveness of pressure regulation, we introduce the pressure differential *P*_D_, defined as the difference between the maximum pressure and the critical threshold, which reflects the available pressure margin above the threshold required for MTRG reset. This metric offers insight into the regulatory capacity provided by each narrow seam level, as depicted in Fig. 5C. Notably, for seams with levels 5 and 6, the pressure *P*_D_ is negative, indicating that the maximum pressure is insufficient to trigger the MTRG reset. Therefore, these levels are deemed unsuitable for reliable operation. Then, the inflation behavior of narrow seams at levels 1 to 4 is further analyzed, with results summarized in Fig. 5D. For example, under level 1, the airbag reaches the critical threshold at *t*_1_ = 5.31 s and achieves maximum pressure at *t*_2_ = 15.56 s. While variations in the level of seams influence the airbag inflation duration, these differences generally remain within a few seconds and do not significantly impact overall performance. However, the selection of the level of seams will play a critical role in determining the passive deflation duration of the airbag.

To evaluate this, we also define *t*_3_ as the time required for the air pressure to drop to 0.50 kPa following deactivation of the air pump—a threshold below which further pressure changes no longer influence the state transition of the MTRG. Among the tested configurations, the absence of seams (level 0) results in an excessively long passive deflation time of 632 s, which severely limits the robotic gripper’s ability to perform high-frequency repetitive grasping (Fig. 5E). In contrast, the introduction of seams greatly reduces *t*_3_, with all tested levels demonstrating at least a 10-fold improvement. Notably, level 3 achieves rapid deflation in only 19.50 s, substantially enhancing the responsiveness of the system. Based on this performance, this level is selected for integration into the MTRG. Under this configuration, the MTRG can be reset within 15 s, and the airbag completes deflation within 20 s, demonstrating its suitability for rapid, repetitive operations, as illustrated in Fig. 5F (Movie S7).

### UAVs perching enabled by MTRGs

**Fig. 6.**
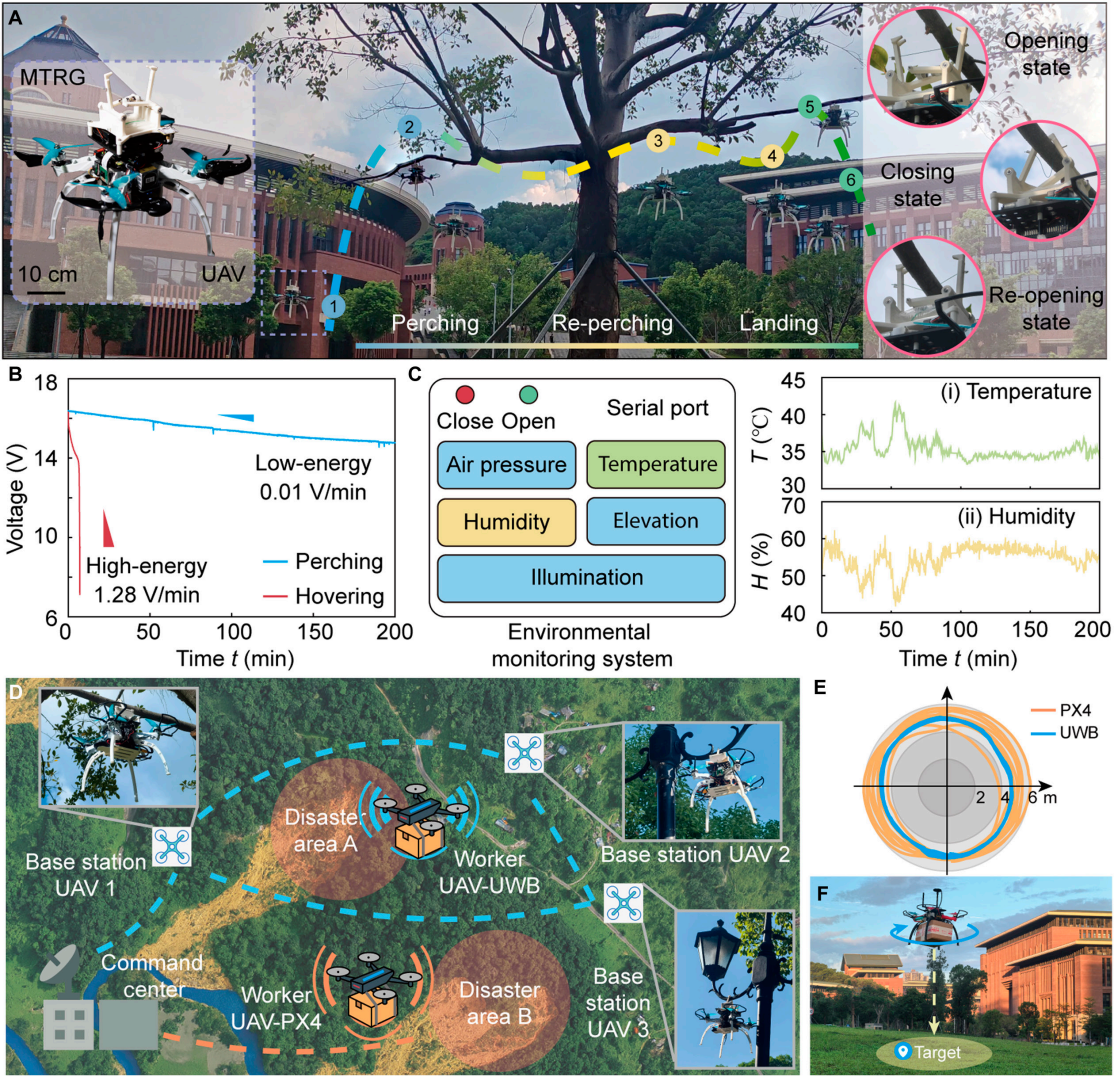
Outdoor experiments demonstrating MTRG-enabled UAV perching. (A) UAVs repeatedly perform perching and takeoff maneuvers from tree branches, with 3 snapshots presented on the right. (B) Comparison of voltage variations when the UAV hovers and perches, respectively. (C) Environmental monitoring such as temperature and humidity utilizing onboard sensors. (D) Three UAVs perched in different locations construct a communication network for improving positioning accuracy. (E) Comparison of positioning accuracy between traditional methods and communication network enhancement methods. (F) Demonstration of object delivery by UAVs.

We integrate our MTRG with a UAV platform to enable stable, low-energy perching, as showcased in Fig. 6A and Fig. S16 (Movie S8). This integration makes the UAV securely attach to surrounding structures and maintain position without continuous energy expenditure on hovering. Through experimental trials, we show the UAV’s ability to repeatedly transition between free flight and perching states, highlighting both the robustness of the MTRG and the repeatability of the system’s operation. Such abilities provide a technical foundation for UAVs to extend flight endurance by actively utilizing their surroundings as functional supports (Fig. 6B and Fig. S17). The experimental results show that the incorporation of the MTRG has a negligible effect on flight performance and attitude stability (Figs. S18 and S19). More importantly, this advancement greatly enables UAVs to dynamically adapt to their operational modes, from aerial mobility to environmental anchoring, thereby expanding the range of tasks they can implement in surveillance, environmental monitoring, and infrastructure interaction (Fig. S20 and Movie S9). To further show the utility of this approach, we equip the UAV with multiple onboard sensors, enabling it to monitor environmental conditions such as temperature and humidity during perching (Fig. 6C). This integration exemplifies how low-energy perching can transform UAVs into versatile and persistent platforms for data collection and situational awareness in complex environments.

In addition, we integrate 3 UAVs with perching capabilities, enabling the construction of a localized and high-precision network. Specifically, these stationary UAVs are outfitted with UWB positioning station modules, forming a mobile infrastructure that offers accurate and reliable location services in environments where traditional signals are degraded or unavailable (Fig. 6D). This distributed aerial station architecture allows the network to adapt dynamically to mission requirements while remaining fully untethered. To evaluate the effectiveness of this method, we conduct a comparative positioning experiment. A test UAV, equipped with both UWB and conventional GPS–inertial measurement unit (IMU) fusion systems, records positioning data from both modalities while implementing 8 repeated circular flight trajectories around a fixed point, as shown in Fig. 6E. The comparative results demonstrate that UWB-based localization achieves substantially higher accuracy than GPS-based positioning, as shown in Fig. S21. GPS trajectories exhibit significant deviations due to signal instability, with instances of temporary positioning failure. UWB localization maintains consistent accuracy throughout the trials, without observed failures. This is attributed to inherent wideband properties of UWB signals, which enable the system to achieve localization precision on the order of 10 cm or better under favorable conditions. In contrast, civilian GPS systems typically showcase errors of several meters, particularly in the cluttered environments. Beyond accuracy, UWB offers the critical advantage of independence from external satellite infrastructure, ensuring robust operation in GPS-denied or degraded conditions, such as indoor environments, urban canyons, or areas with significant signal occlusion. This positioning capability helps to achieve precise object delivery at the designated point, as showcased in Fig. 6F. Furthermore, as the entire UWB infrastructure is carried by UAVs themselves, the system shows a high degree of operational autonomy and adaptability. This feature provides significant advantages for mission scenarios requiring high-reliability, high-precision navigation, such as search and rescue operations, inspection tasks in constrained spaces, and autonomous multi-robot coordination in complex or unstructured environments.

## Discussion

Recent advances in UAVs have always highlighted the critical demands for advanced perching strategies, particularly through the incorporation of bistable robotic grippers [38,45,46]. Such systems allow the UAVs to transition from energy-intensive hovering modes to low-power, stable resting states [20]. Despite their potential, enabling UAVs to implement perching behaviors that are gentle and robust remains a substantial challenge. This limitation arises as energy barriers remain constant in existing bistable mechanisms. At present, the incorporation of active actuators offers a possible solution to overcome this restriction [16]; however, this compromises the inherent passive adaptability of bistable systems, increasing system complexity and energy demand.

In this work, we develop magnetic tensegrity-enabled bistable structures that achieve adaptive energy barriers passively during state transitions, enhancing the adaptability of bistable robotic grippers. Leveraging this mechanism, our MTRG shows compliant yet powerful grasping behaviors (0.12 N for triggering and 35.05 N for grasping), accomplishing a force ratio of up to 300, a performance level comparable to natural grasping capabilities exhibited by bats. Our artificial robotic gripper is a great example of the concept of mechanical intelligence (MI), which integrates intelligence directly into structural design, i.e., leveraging mechanical systems to perform tasks without requiring a complex control system [47–50]. When integrated with UAVs, this MI-based robotic gripper substantially enhances aerial perching and manipulation abilities. Specifically, it allows UAVs to securely perch for extended durations, minimizing the need for continuous hovering and extending operational standby periods through lower energy consumption. Beyond energy efficiency, the ability to perch reliably across diverse environments opens pathways for long-term stable functions, where UAVs can act as persistent, adaptive platforms. Moreover, by incorporating multiple UAVs with perching capability, a low-cost, reconfigurable, and mobile communication network for local and remote areas can be constructed. Such networks support demands in environmental monitoring, disaster response, and temporary infrastructure deployment, highlighting the broader potential of magnetic tensegrity-based bistable mechanisms for advancing the functionality and versatility of UAVs.

While the current paradigm of our robotic gripper shows encouraging experimental outcomes, several limitations remain that must be systematically addressed before real-world deployment is realized. First, the geometry of the fingers has not yet undergone optimization. Future designs should further utilize diverse analysis approaches, such as computational modeling, evolutionary algorithms, and finite element analysis, to yield a configuration that can maximize force distribution and contact reliability. Second, a primary technical challenge arises from the use of magnetic elements within the robotic gripper. This introduces the potential for interference with onboard sensors, such as magnetometers and IMUs. Future work will explore the incorporation of advanced materials, shielding techniques, or compensation algorithms specifically designed to reduce electromagnetic interference. Lastly, the current application scenario does not fully reflect real-world conditions. In future work, it will be necessary to conduct experimental evaluations in more unconventional and complex environments to validate the system’s robustness and applicability.

## Data Availability

The authors declare that the main data supporting the findings of this study are available within the article.

## Supplementary Materials

Supplementary 1 Supplementary Text Notes S1 to S4 Figs. S1 to S21 Tables S1 to S3 Movies S1 to S9
