# Supplementary material for: Magnetic Tensegrity-Enabled Robotic Gripper with Adaptive Energy Barrier for UAV Perching
Source: Cyborg Bionic Syst. 2026 Mar 9;7:0535. doi: 10.34133/cbsystems.0535 (PMC12968396; doi:10.34133/cbsystems.0535)
Supplement: Supplementary 1 — Supplementary Text Notes S1 to S4 Figs. S1 to S21 Tables S1 to S3 Movies S1 to S9 [file cbsystems.0535.f1.zip › Supplementary Information.docx]

Supplementary Information for

**Magnetic tensegrity-enabled robotic gripper with adaptive energy barrier for UAV perching**

**Authors:**

Lulu Han, Hao Yang, Luobin Wang, Yuquan Zheng, Jingrui Yang, Yuxuan Fu, Jieliang Zhao, Zhong Wan, Zhigang Wu ^*^, Jie Zhang ^*^, Jianing Wu ^*^

*Corresponding author E-mail:

[wuzhigang@mail.sysu.edu.cn](mailto:wuzhigang@mail.sysu.edu.cn) (Zhigang Wu)

[zhangj696@dlut.edu.cn](mailto:zhangj696@dlut.edu.cn) (Jie Zhang)

[wujn27@mail.sysu.edu.cn](mailto:wujn27@mail.sysu.edu.cn) (Jianing Wu)

**The file includes:**

Supplementary Text

Note S1 to S4

Figures S1 to S21

Tables S1 to S3

Video S1 to S9

References

**Note S1. Relationship between force and distance of coaxial cylindrical magnets**

The calculation of the magnetic force between cylindrical magnets can be performed using the simplified model [S1]. The magnets are defined as follows: two cylindrical magnets with radii $r_{1}$ and $r_{2}$ are aligned coaxially, with opposite poles facing each other, as shown in Fig. S1. The axial positions of the corresponding end faces are represented as $z_{1}$, $z_{2}$, $z_{3}$, and $z_{4}$. The magnetization intensities of the two magnets are represented by $J_{1}$ and $J_{2}$, and the vacuum permeability is denoted by *μ*_0_.

When the cylindrical magnets are coaxially aligned with opposite poles facing each other, the axial attractive force *F*_z_ can be expressed as follows.

|  | $F_{z}=\frac{J_{1}J_{2}}{2\mu_{0}}\sum_{i=1}^{2} \sum_{j=3}^{4} a_{1}a_{2}a_{3}f_{z}^{'}\left[ -1 \right]^{i+j}$ | (S1) |
| --- | --- | --- |

where,

|  | $f_{z}^{'}=K\left( a_{4} \right)-\frac{1}{a_{2}}E\left( a_{4} \right)+\left[ \frac{a_{1}^{2}}{a_{3}^{2}}-1 \right]\Pi\left( \frac{a_{4}}{1-a_{2}}\vert a_{4} \right)$ | (S2) |
| --- | --- | --- |
|  | $a_{1}=z_{i}-z_{j}$ | (S3) |
|  | $a_{2}=\frac{[r_{1}-r_{2}]^{2}}{a_{1}^{2}}+1$ | (S4) |
|  | $a_{3}^{2}=\left[ r_{1}+r_{2} \right]^{2}+a_{1}^{2}$ | (S5) |
|  | $a_{4}=\frac{4r_{1}r_{2}}{a_{3}^{2}},0<a_{4}\leq1$ | (S6) |

In the simplified calculation formula, two cases may lead to singularities. The first case occurs when the radii of the two magnets are equal, where $a_{2}=1$, resulting in a zero denominator in the first argument of the $\Pi\left( \frac{a_{4}}{1-a_{2}}|a_{4} \right)$ term. In this situation, $\text{Π}\left( \text{±∞|}\text{a}_{\text{4}} \right)\text{=0}$ can be directly applied. The second case arises when the two magnets are in complete contact, i.e.,$z_{2}=z_{3}$. During computation, this condition yields $a_{1}=0$, which in turn causes the denominator of $a_{2}$ to vanish. Since no force component is generated between coincident surfaces, when $a_{1}=0$, the corresponding $a_{1}a_{2}a_{3}f_{z}^{'}$ term in the summation of Eq. (S1) should be set to zero, while all other terms are calculated as usual.

When the two magnets are identical, i.e., $J_{1}=J_{2}=J$ , $r_{1}=r_{2}=r$ , $z_{1}-z_{3}=-L-d$ , $z_{1}-z_{4}=-2L-d$ , $z_{2}-z_{3}=-d$ and $z_{2}-z_{4}=-L-d$ ,where $L$ is the thickness of the magnet and $d$ is the gap between the magnets, then $a_{2}=1$.According to the definition of the complete elliptic integral of the third kind $\text{Π}\left( \text{±∞|}\text{a}_{\text{4}} \right)\text{=0}$, the $\left[ \frac{a_{1}^{2}}{a_{3}^{2}}-1 \right]\Pi\left( \frac{a_{4}}{1-a_{2}}|a_{4} \right)$ term in Eq. (S2) becomes zero, and the calculation formula can be simplified to the following form:

|  | $F_{z}=\frac{J^{2}}{2\mu_{0}}\sum_{i=1}^{2} \sum_{j=3}^{4} a_{1}a_{3}f_{z}^{'}\left[ -1 \right]^{i+j}$ | (S7) |
| --- | --- | --- |
|  | $f_{z}^{'}=K\left( a_{4} \right)-E\left( a_{4} \right)$ | (S8) |
|  | $a_{1}=z_{i}-z_{j}$ | (S9) |
|  | $a_{3}^{2}=4r^{2}+a_{1}^{2}$ | (S10) |
|  | $a_{4}=\frac{4r^{2}}{a_{3}^{2}},0<a_{4}\leq1$ | (S11) |

**Note S2.** **Stability analysis of the magnet pairs**

In this study, the bistable structure is constructed by leveraging the attractive force between a pair of permanent magnets. To ensure stability, it is necessary to analyze the conditions under which the two magnets can maintain mutual attraction. The magnet pair can be simplified as the configuration illustrated in Fig. S4A, where the two magnets are hinged to fixed boundaries on opposite sides. Each magnet is subjected to gravity, the attractive magnetic force from the counterpart, and the reaction force at the hinge, with the forces confined to the plane of the figure. At the illustrated position, the effect of gravity is maximal; thus, analyzing stability at this configuration is sufficient to guarantee stability under other conditions. Given the complexity of the exact magnetic model, an experimental method is employed to determine the maximum separation distance at which the magnets maintain stable attraction. In the experiment, the two magnets are hinged and mounted on bases aligned with a linear slide rail (Fig. S4B). The left base is fixed, while the right base is gradually moved outward from the position where the magnets are just in contact. The displacement of the right base at the moment when the magnets can no longer remain attracted due to gravity is recorded as the maximum distance for stable attraction. This distance defines the design limit, within which the structure of the gripper must be constrained.

As shown in Fig. S4C, when the distance between the magnet pair is set to 10 mm, they exhibit high coaxial alignment, ensuring stable attraction. When the distance increases to 20 mm, the magnets remain mutually attractive but display slight tilting. At 27 mm, which is close to the critical value, the magnets tilt excessively and the attractive force becomes weak, such that even small perturbations can break the attraction. Overall, the experiment demonstrates that a distance within 20 mm is sufficient to ensure stable operation of the mechanism, while beyond 27 mm the magnets can no longer maintain attraction. In this study, the maximum separation distance of the magnet pair is ~10 mm, which fully satisfies the requirement for stable mutual attraction.

**Note S3. Static equilibrium equations of the robotic gripper**

Assuming the gripper is subject to symmetric forces, and referring to Fig. 2A, the potential energy of the gripper system at a joint angle $\theta$ is determined by the distance between points $P$ and $Q$. According to geometric relationships, this distance can be expressed as:

|  | $\overline{PQ}=2\overline{AB}\sin\theta+2\overline{BQ}\cos\theta-\overline{AA'}$ | (S12) |
| --- | --- | --- |

where, $\text{A}\text{'}$ is the symmetric point of $\text{A}$.

Assuming the attractive force between the two magnets is sufficiently strong and gravity is negligible, and that the magnets are perfectly aligned and attract each other directly, the distance between the magnets is: $\text{d}\text{=}\overline{\text{PQ}}\text{-2}\text{L}$, in which $L$ represents the thickness of a single magnet. Consequently, the magnetic attractive force $F_{M}$ can be written as:

|  | $\text{F}_{\text{M}}\text{=}\text{F}_{\text{z}}\text{(}\text{d}\text{, r, J}\text{)}$ | (S13) |
| --- | --- | --- |

where $\text{F}_{\text{z}}()$ represents a nonlinear function that describes the magnetic force as a function of distance, which can be evaluated using Eq. (S7). Here, $r$ represents the radius of the cylindrical magnet, while $J$ represents its magnetization intensity. Since the magnetic force model $F_{z}()$ involves elliptic integrals, its direct evaluation is computationally expensive and leads to long numerical analysis times. Therefore, for batch computations, the model is first numerically fitted over a specified range. Based on empirical observations, the force–distance relationship can be well approximated by $\text{F}_{\text{M}}=C_{1}/{(d+C_{2})}^{2}$ which provides a good fitting accuracy.

Performing a force analysis on a single (left) finger of the gripper, at joint angle $\theta$ and assuming no external force acts at joint $A$ (gravity is neglected), the torque $M$ at joint $A$ is given by:

|  | $\text{M}\text{=}\text{F}_{\text{M}}\overline{\text{BQ}}\sin\text{θ}\text{-}\text{F}_{\text{M}}\overline{\text{AB}}\cos\text{θ}$ | (S14) |
| --- | --- | --- |

At the critical point of bistable switching, the system satisfies:

|  | $M_{trigger}=0$ | (S15) |
| --- | --- | --- |

Solving this equation yields:

|  | $\text{θ}_{\text{trigger}}\text{=arc}\tan\frac{\overline{\text{AB}}}{\overline{\text{BQ}}}$ | (S16) |
| --- | --- | --- |

Thus, the critical switching angle depends solely on the ratio between $\overline{AB}$ and $\overline{BQ}$. This angle characterizes the sensitivity of the bistable transition: the larger the critical angle, the less sensitive the gripper is to actuation, requiring a greater deformation to trigger switching.

In the design of the gripper, we assume that $\overline{AA'}$, $\overline{AD}$, and $L$ are fixed. The maximum closing angle of the gripper is set to 30°, and cylindrical neodymium magnets with a diameter and length of 10 mm are used. Based on the magnetic force-displacement curve of the selected magnets, the distance $d$ between the magnets must remain within the range $[0,10\mathrm{mm}]$; beyond this range, gravitational effects dominate and stable magnetic attraction cannot be guaranteed. The remaining design parameters $\overline{AB}$ and $\overline{BQ}$ are subject to optimization.

**Note S4: Cyclic test experiment**

The cyclic test system is composed of the gripper, a force sensor, an optical sensor, an airbag, an air pump, a solenoid valve, a control board (Arduino Uno), a display screen, and a fixed base (Fig. S6) The gripper body serves as the test subject, while the experiment aims to evaluate the reliability of the gripper and the airbag recovery system, ensuring long-term operational stability. The force sensor is employed to provide a stable triggering condition and to measure the contact force during the triggering process. An optical sensor is mounted on the gripper base, equipped with an infrared transmitter–receiver pair. A thin blocking plate is fixed to the bottom of the gripper’s finger and rotated together with it. The relative positions of the blocking plate and optical sensor are adjusted so that, in the open state, the plate consistently blocks the infrared beam, while in the closed state, the beam is unblocked. Thus, the optical sensor output corresponds directly to the open (blocked) and closed (unblocked) states of the gripper. The solenoid valve is a normally closed three-way type. In the absence of input voltage, the valve connects the airbag to the external environment, allowing deflation. With a 5 V input, the valve connects the airbag to the air pump for inflation. The control board determines the gripper state based on the optical sensor output: in the open state, both the solenoid valve and air pump are supplied with 0 V, while in the closed state, both are supplied with 5 V. The display screen is used to present system status, and the fixed base secures the gripper and auxiliary components.

The cyclic experiment begins with the robotic gripper in the open state. The force sensor moves downward at a constant speed from a designated initial position until it contacts the trigger rope of the robotic gripper, recording the contact force during the process. Upon reaching a specified displacement, the gripper is triggered and transitioned to the closed state. At this point, the optical sensor becomes unblocked, outputting a high-level signal to control boards, which in turn activates both the solenoid valve and the air pump to inflate the airbag for resetting. Once the reset process begins, the force sensor is lifted back to its initial position. Meanwhile, the air pump continues to inflate the airbag for 4 s (since passive venting through the narrow seam is not used in the cyclic test, this duration is sufficient for reset). Afterward, the gripper reopens and control systems return to its initial state, with the solenoid valve and pump deactivates and airbags vent to the environment. At this point, the system is fully restored and ready for the next triggering cycle. This process is repeated for a total of 1000 cycles, after which the system automatically stops.


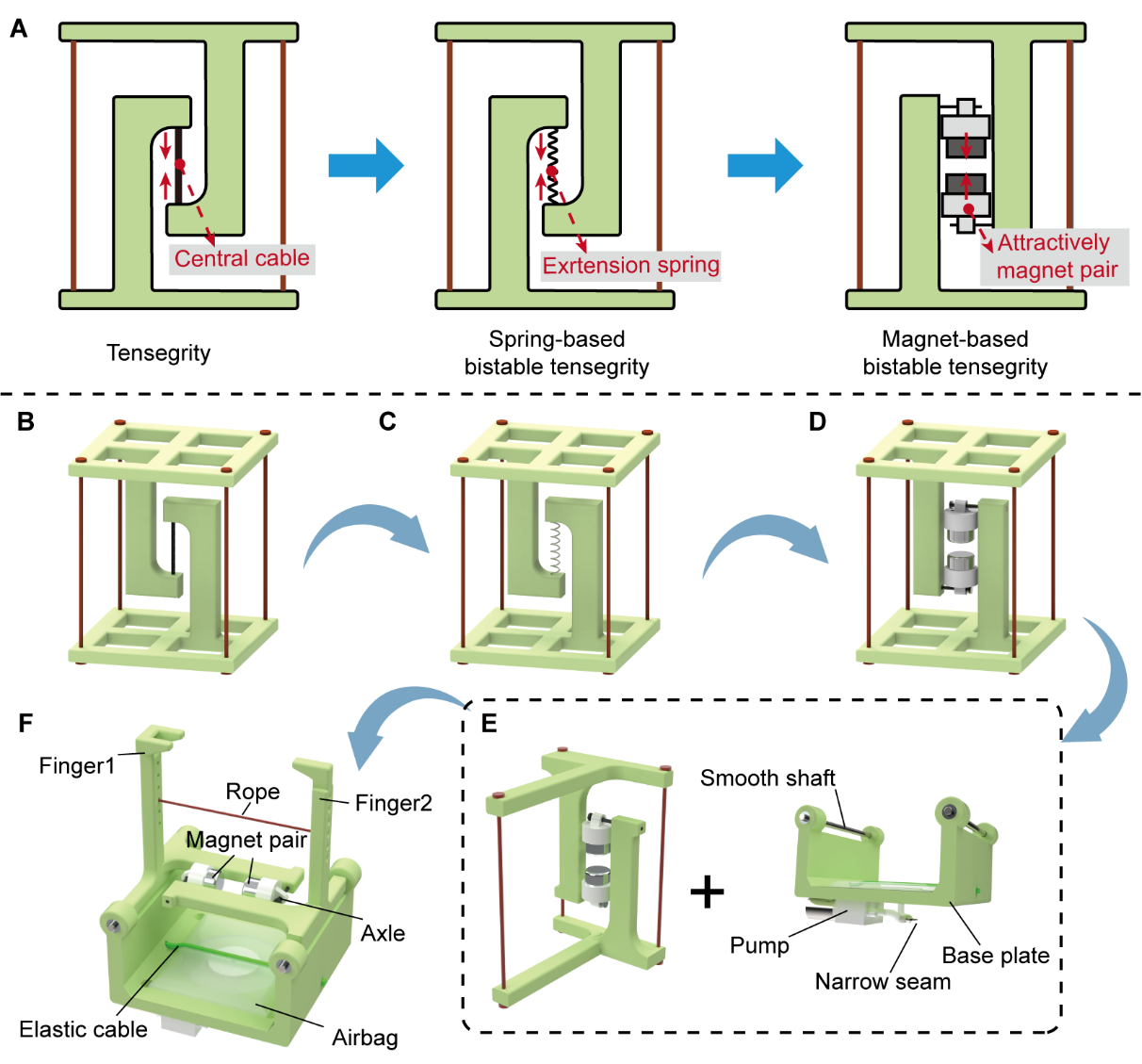


**Fig. S1.** Design process of MTRGs. (A) The design process of our magnetic tensegrity structure (B) Classical anti-gravity tensegrity structure. (C) Bistable tensegrity structure. (D) Magnetic tensegrity structure. (E) Main components of MTRGs. (F) Configuration of MTRGs.

**
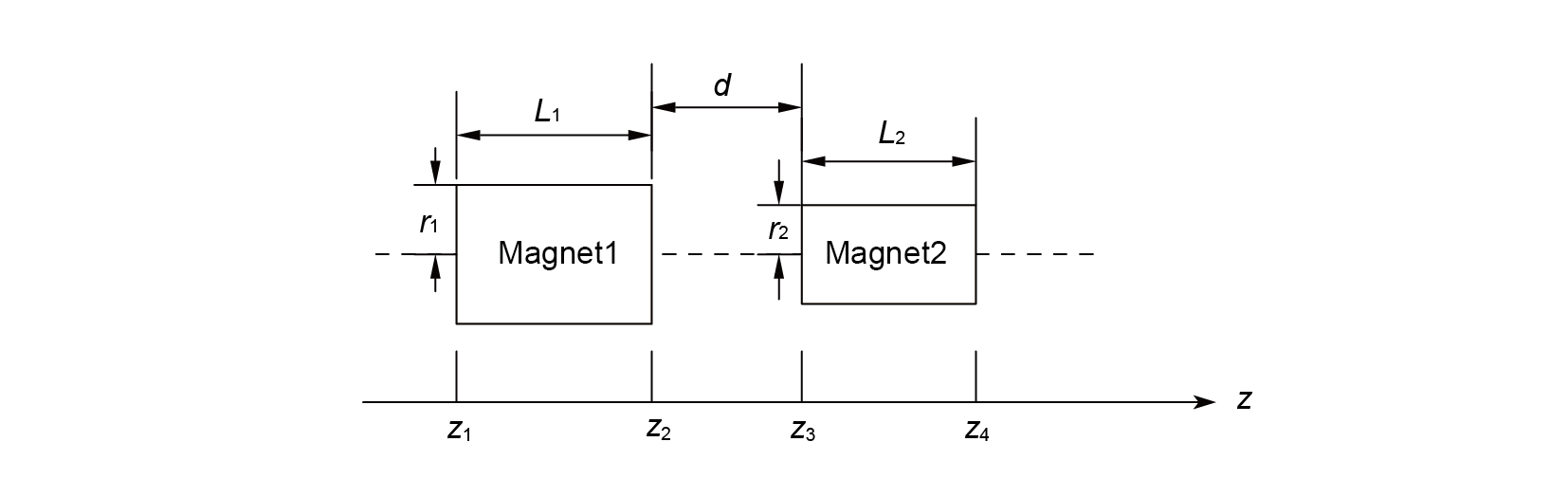
**

**Fig. S2.** Two-dimensional side view of the system composed of two coaxial cylindrical magnets with a generated force on the second magnet.


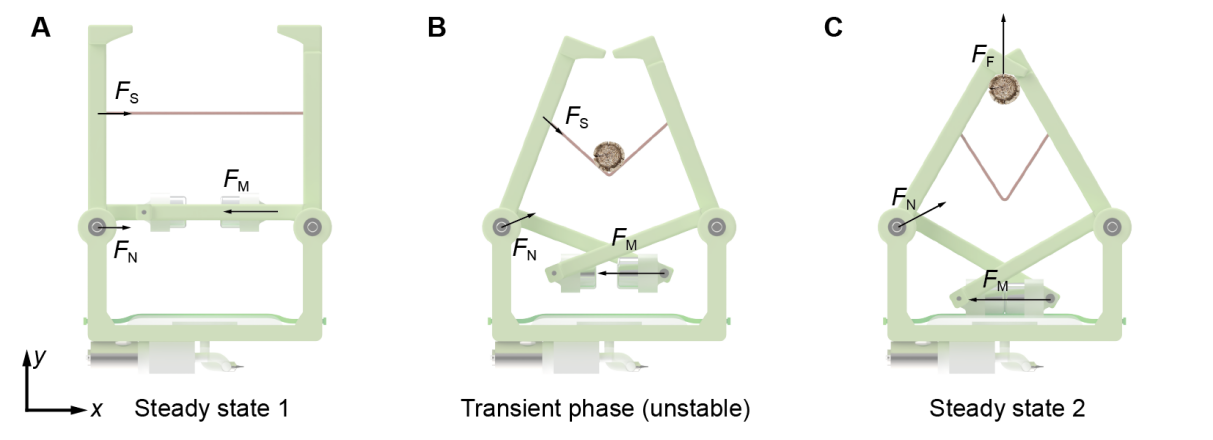


**Fig. S3.** Force diagrams of MTRGs at diverse stages, including (A) stable state 1, (B) transient phase, and (C) stable state 2.

**
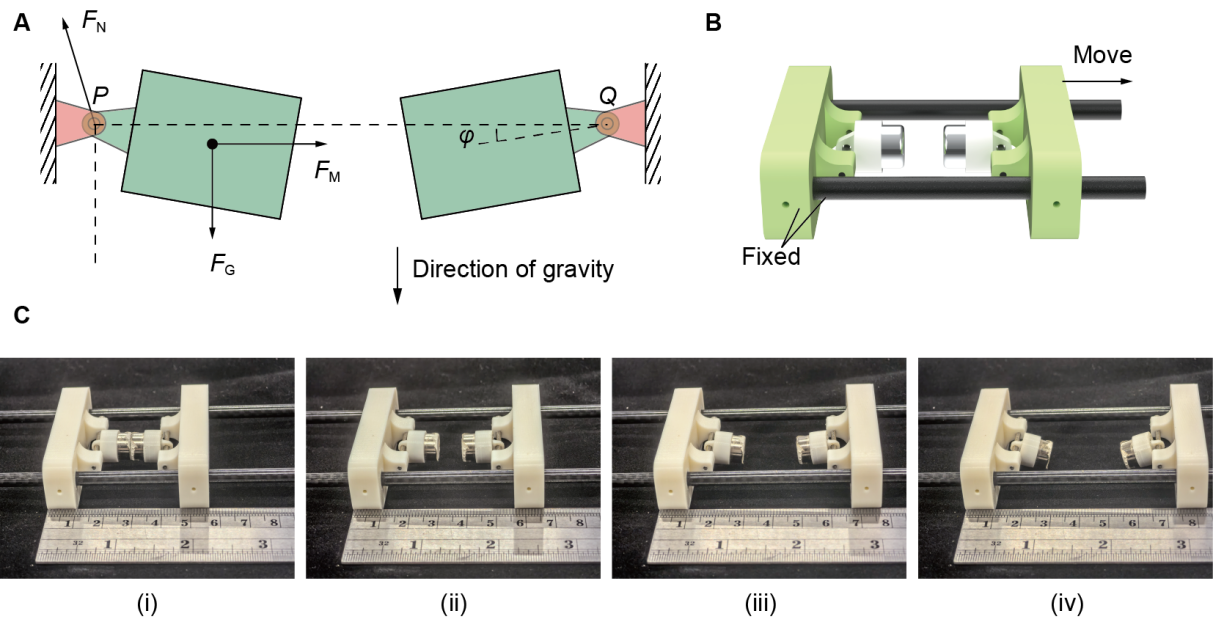
**

**Fig. S4.** Stability analysis of magnet pairs. (A) Force analysis diagram of the magnet pair. (B) Diagram of an experiment to determine the maximum distance of magnet pairs that maintain an attractive state under the influence of gravity. (C) Experimental images: (i) initial state without a gap, (ii) magnet pair showing high coaxial alignment at a 10 mm distance, (iii) when the distance is 20 mm, the magnet pair still remains mutually attractive with slight tilting, and (iv) the snapshot is recorded when the distance is 27 mm.


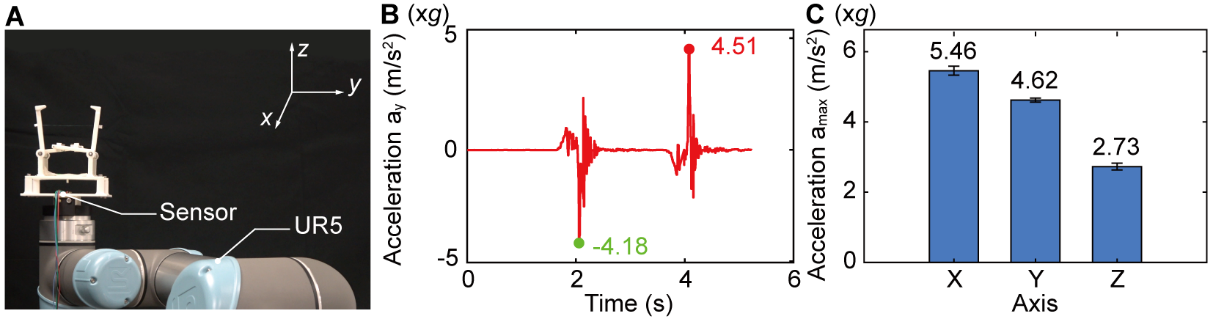


**Fig. S5.** Maximum acceleration required for the state transition of the robotic gripper. (A) Experimental setup. (B) Acceleration of robotic grippers mounted on rigid robotic arm. (C) Maximum acceleration caused by accidental triggering of our robotic gripper.


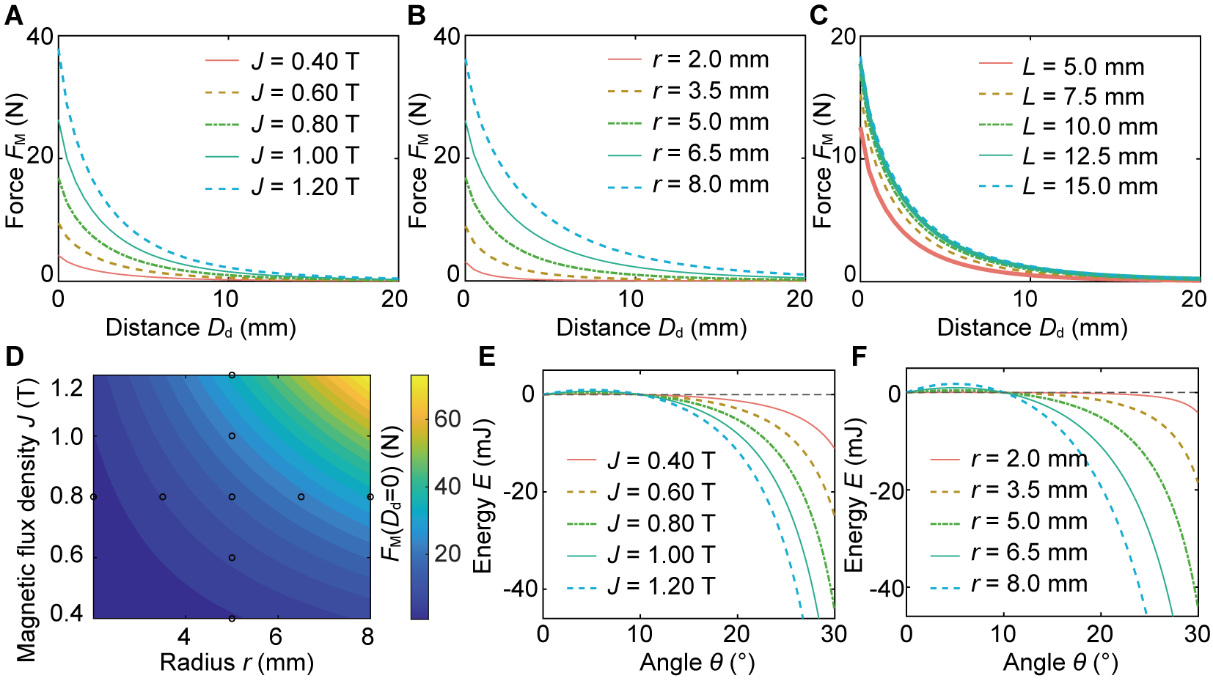


**Fig. S6.** Magnetic force and elastic energy of our robotic gripper. The influence of (A) magnet flux density *J*, (B) magnet radius *r*, (C) magnet length *L* on magnetic force *F*_M_. (D) The magnet force *F*_M_ influenced by radius *r* and magnetic flux density *J*. The effects of (E) magnet flux density *J* and (F) magnet radius *r* on elastic energy *E*.


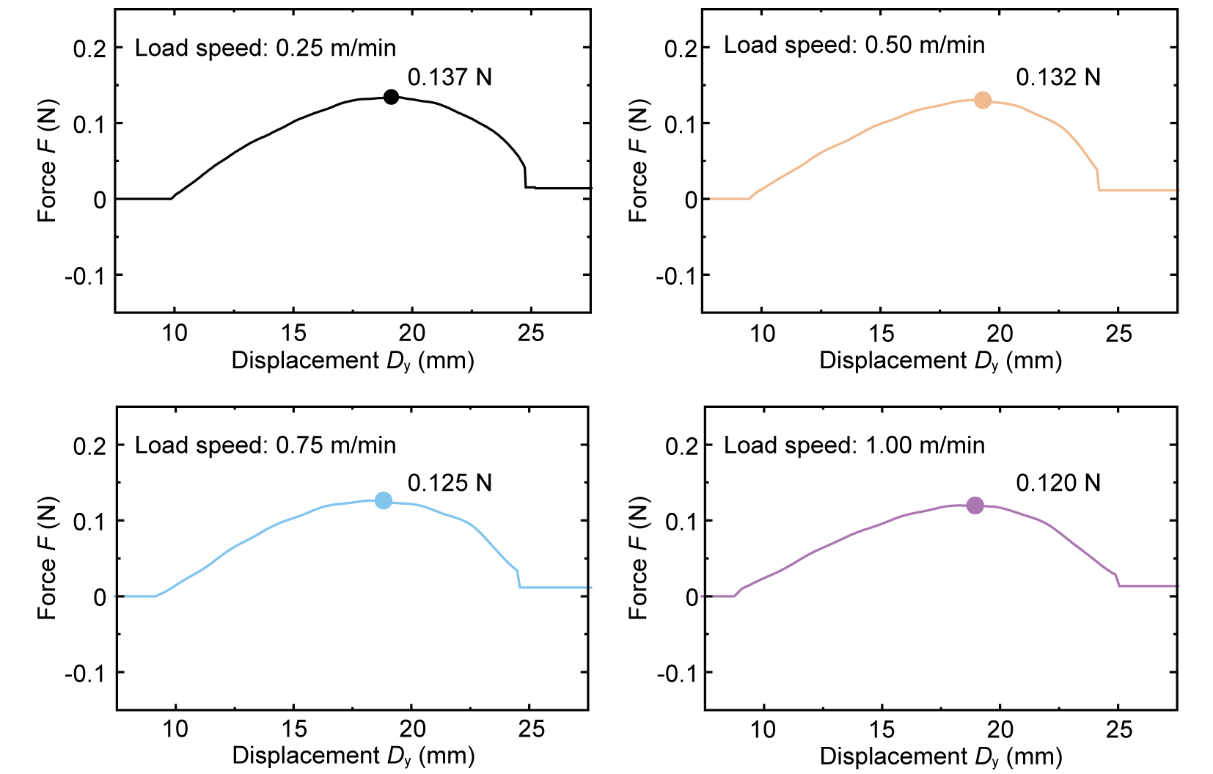


**Fig. S7.** The influence of load contact speed, ranging from 0.25 m/s to 1.00 m/s, on the maximum triggering force.


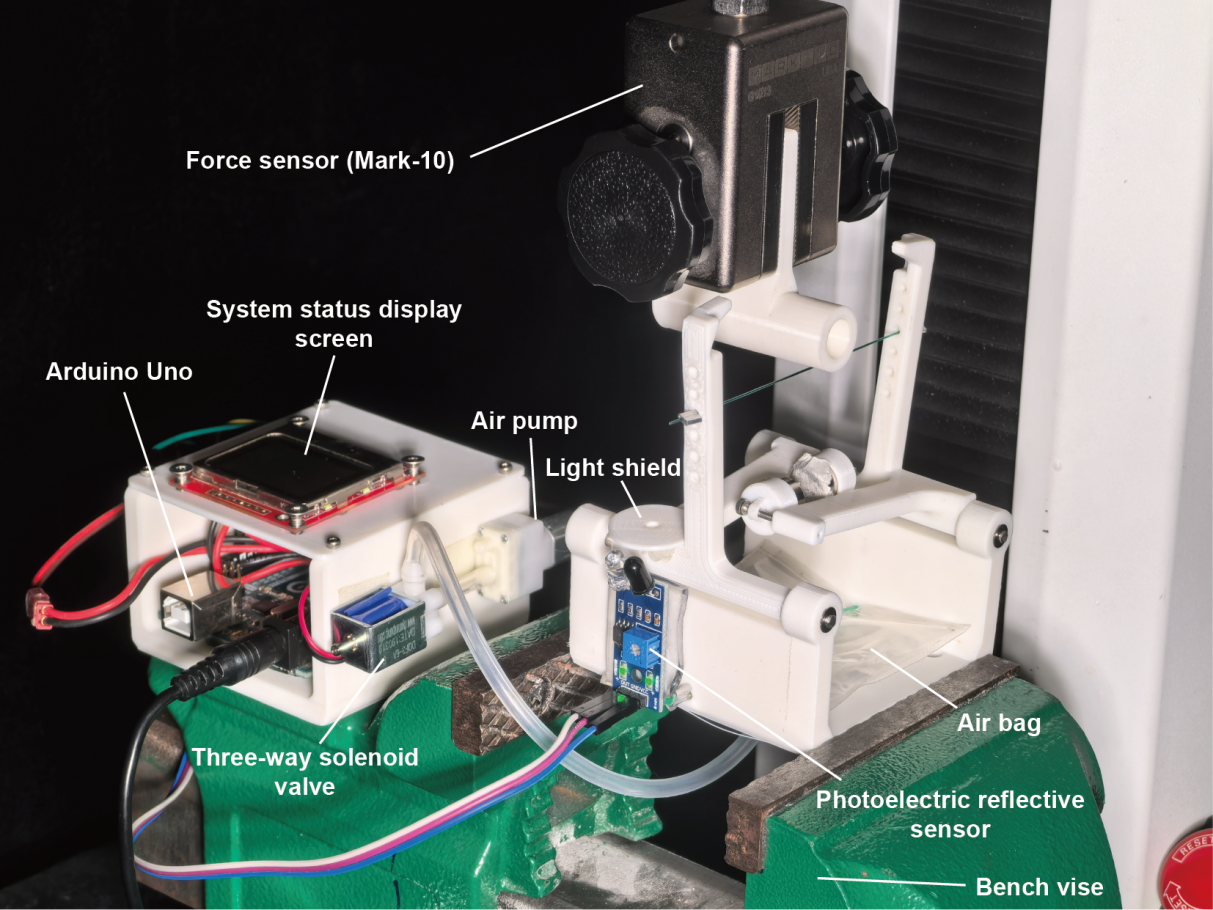


**Fig. S8.** Diagram of circulating experimental system.


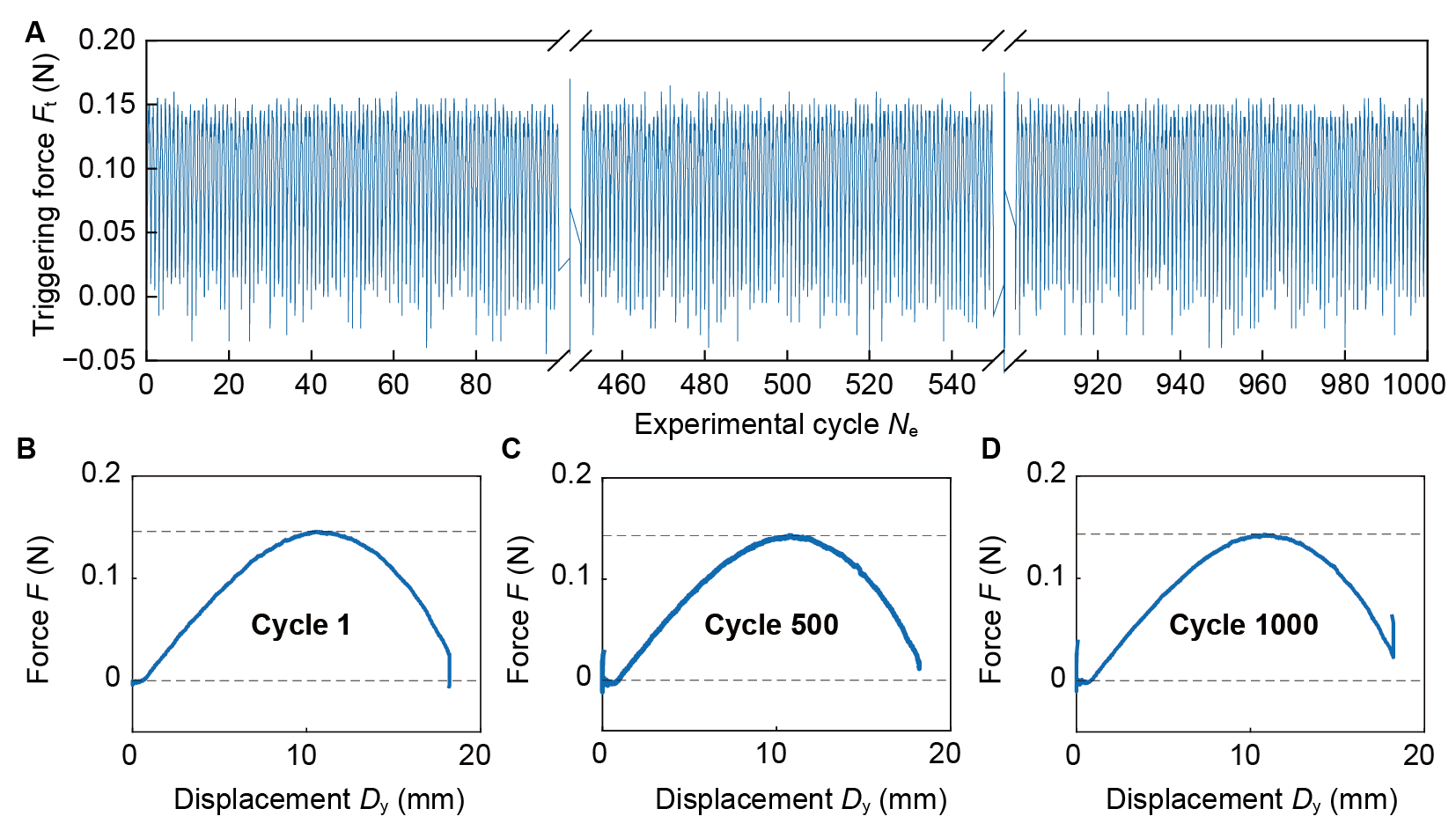


**Fig. S9.** Circulating experimental result. (A) Curve of the triggering force *F*_t_ in 1000 cycles. (B-D) Curves of the 1^st^ ,500^th^, and 1,000^th^ experiments


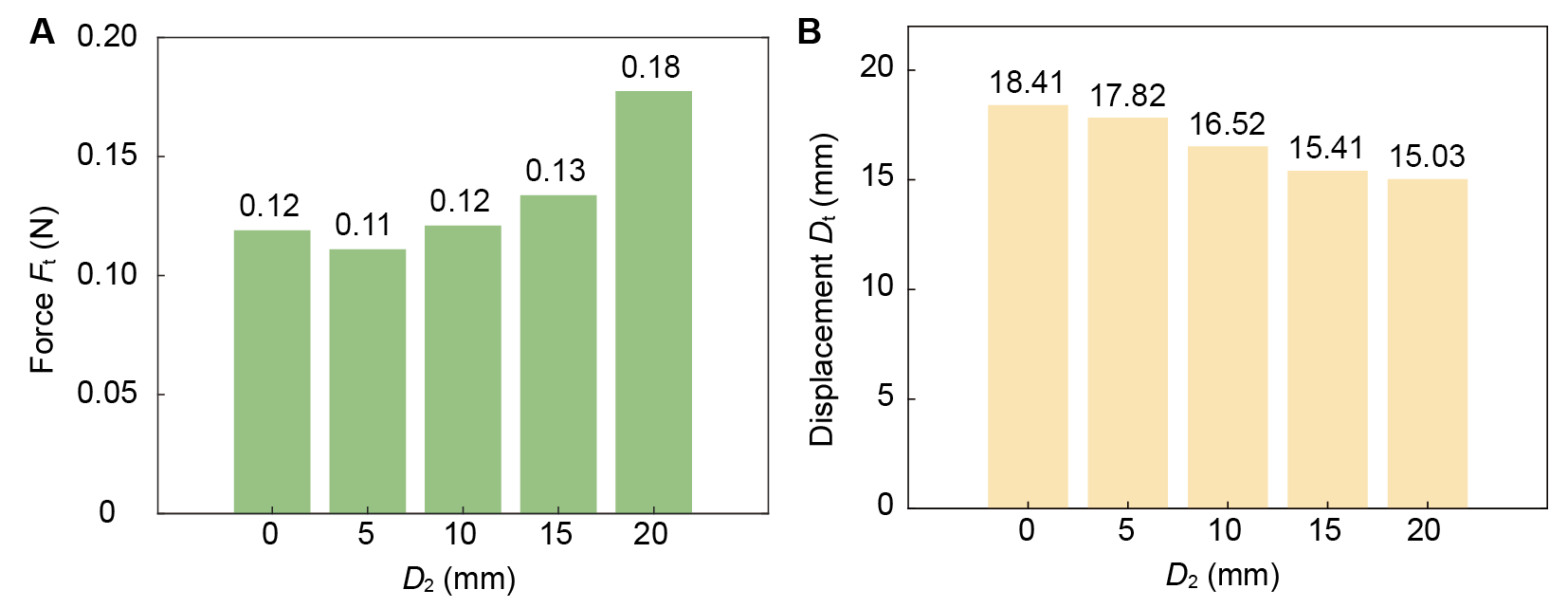


**Fig. S10.** Effect of the trigger position *D*_2_ on (A) triggering force *F*_t_ and (B) triggering displacement *D*_t_.


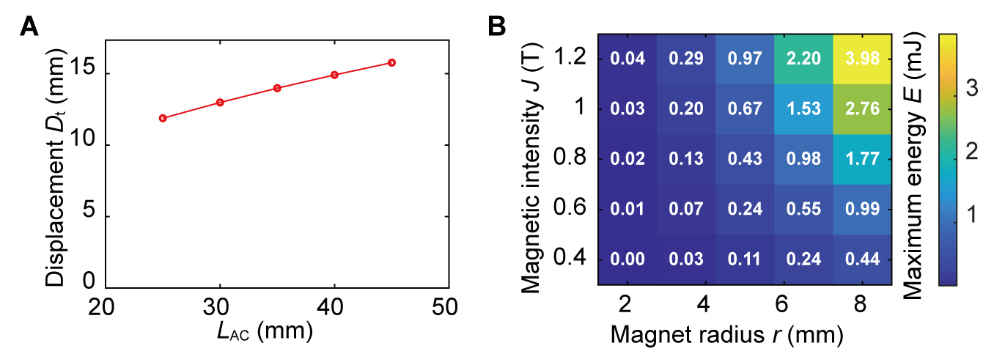


**Fig. S11.** Sensitivity analysis of our robotic gripper. (A) The effects of length *L*_AC_ on triggering displacement *D*_t_. (B) The effects of magnet radius *r* and magnet intensity *J* on maximum energy *E*.


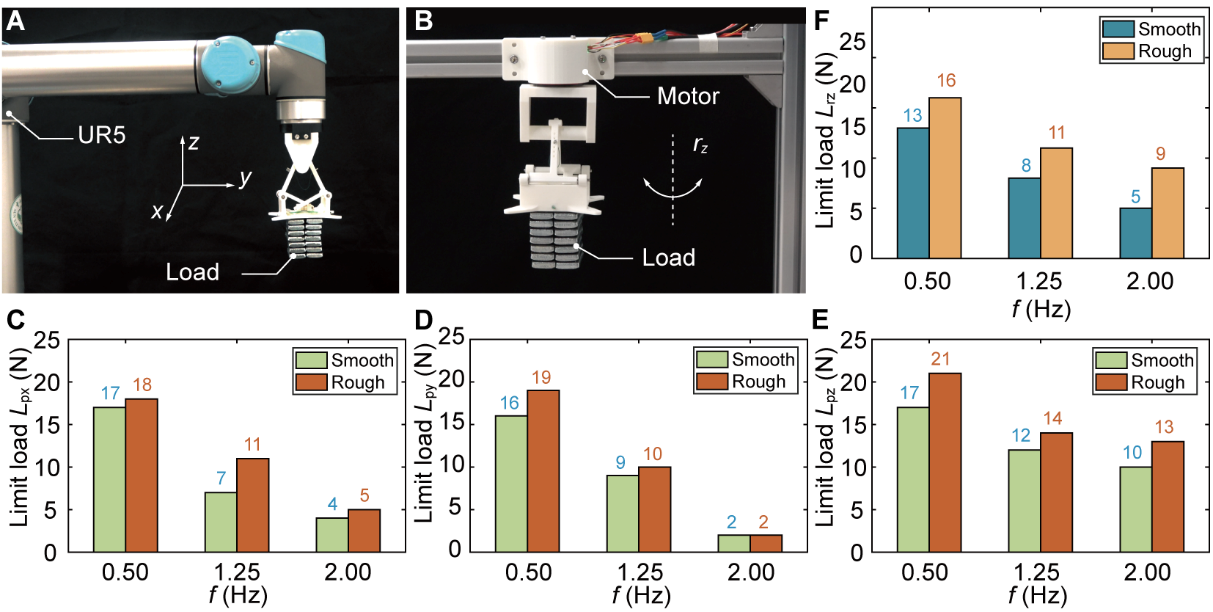


**Fig. S12.** The effects of vibration on grasping stability. Experimental setup for evaluating (A) lateral and (B) torsional loads. (C)-(E) The limit load of the robotic gripper during dynamic motion at different frequencies and in multiple directions. (F) The maximum load when the robotic gripper rotates around the *Z*-axis.


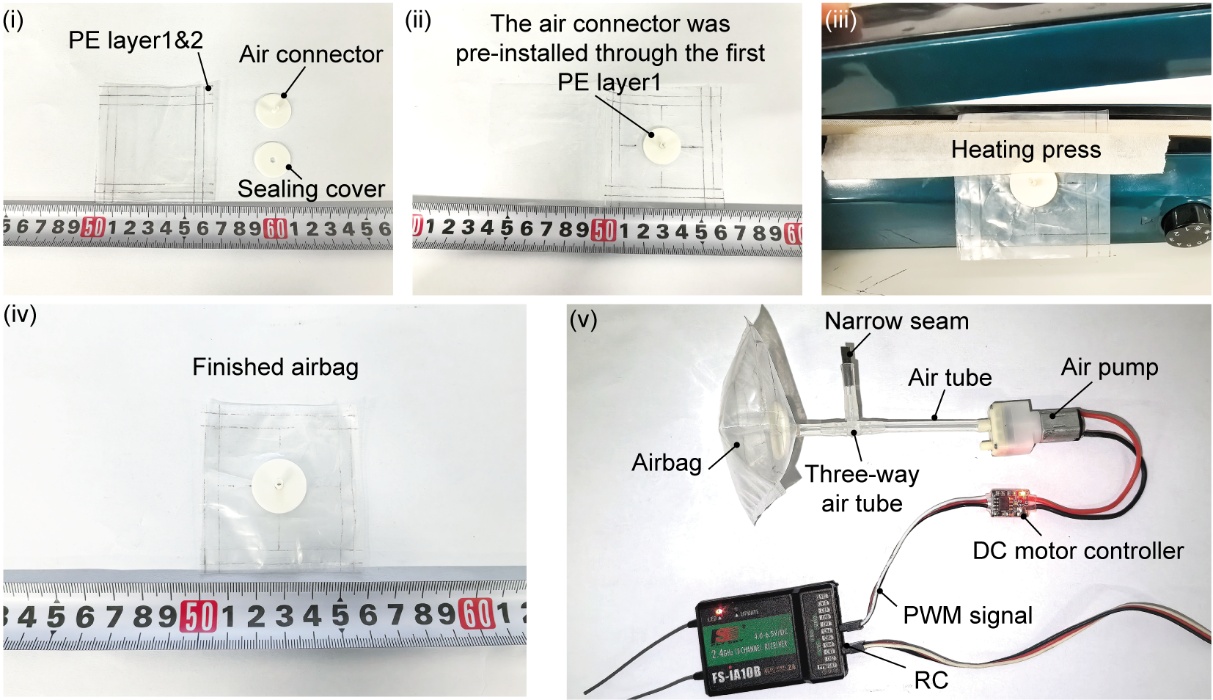


**Fig. S13.** Fabrication process and control strategy of the airbag.


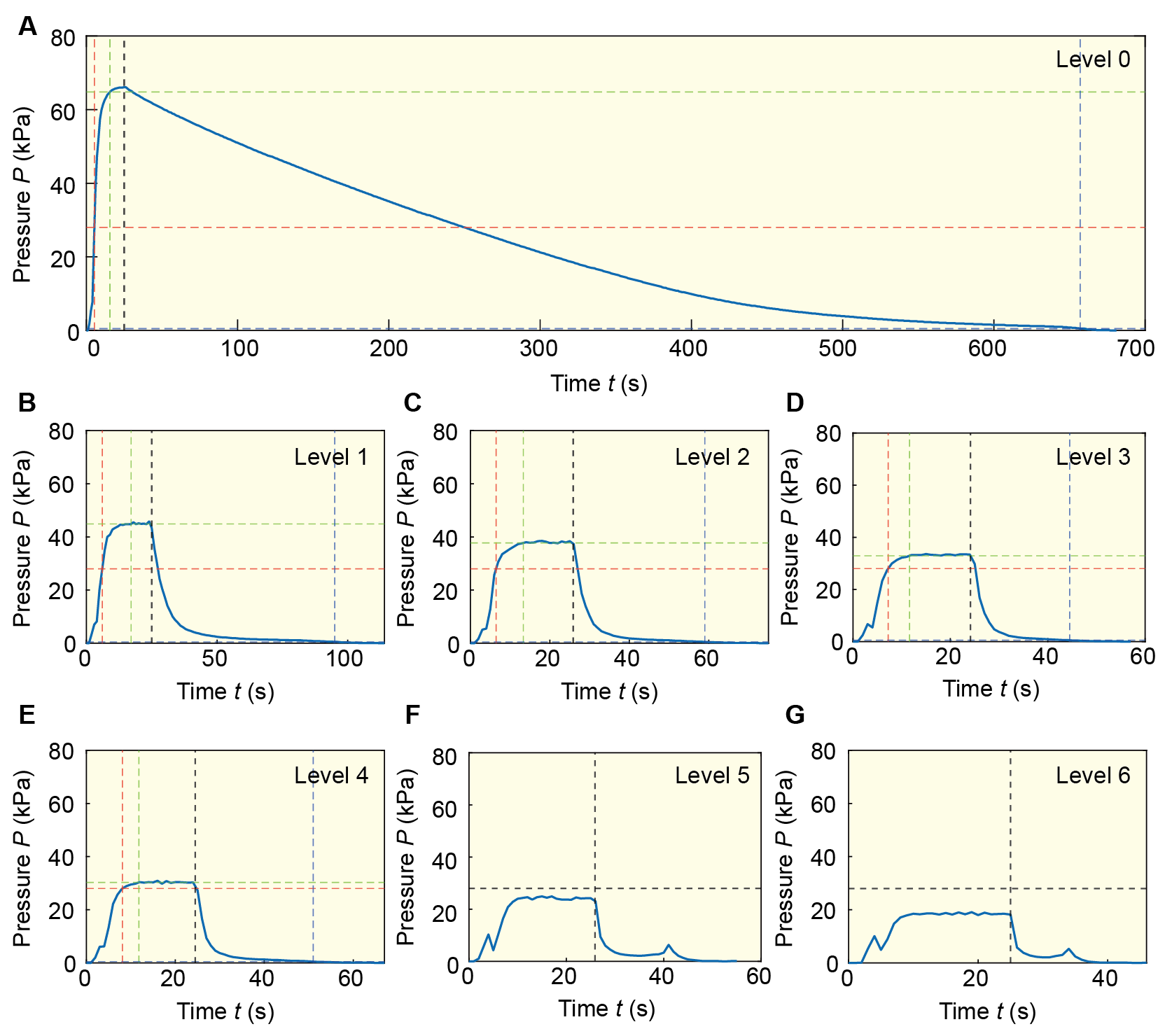


**Fig. S14.** Inflation and deflation pressure curve of narrow seams. (A-G) represent the corresponding results of levels 0-6.


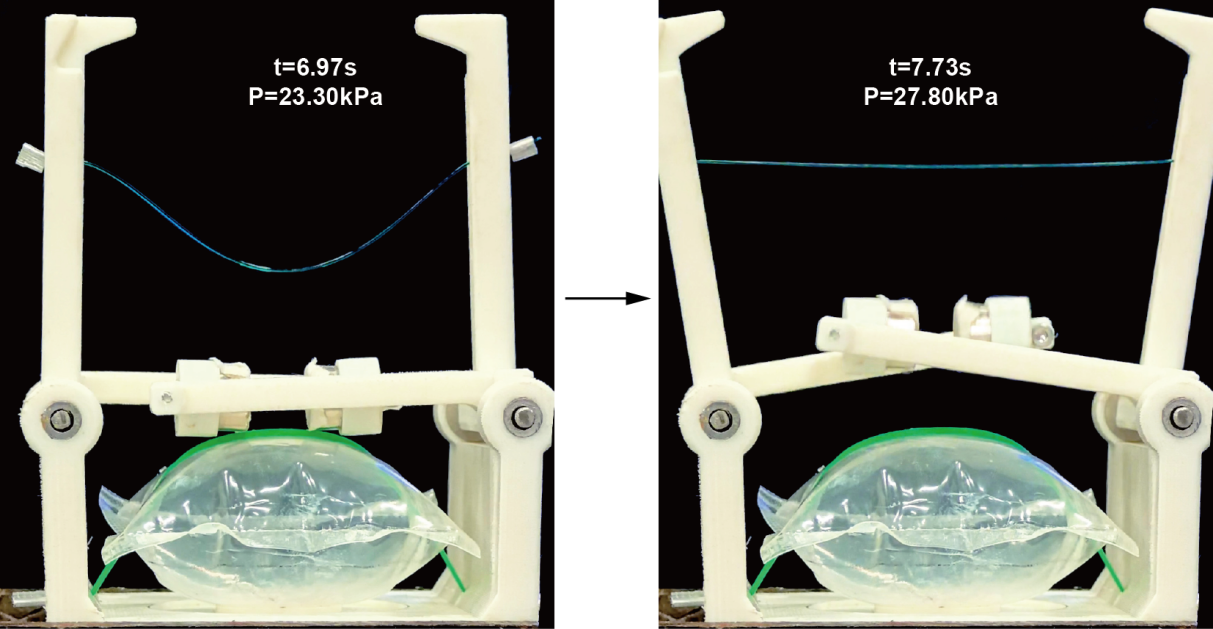


**Fig. S15.** The air pressure diagram at the moment of robotic gripper recovery, illustrated using level 3 as an example.


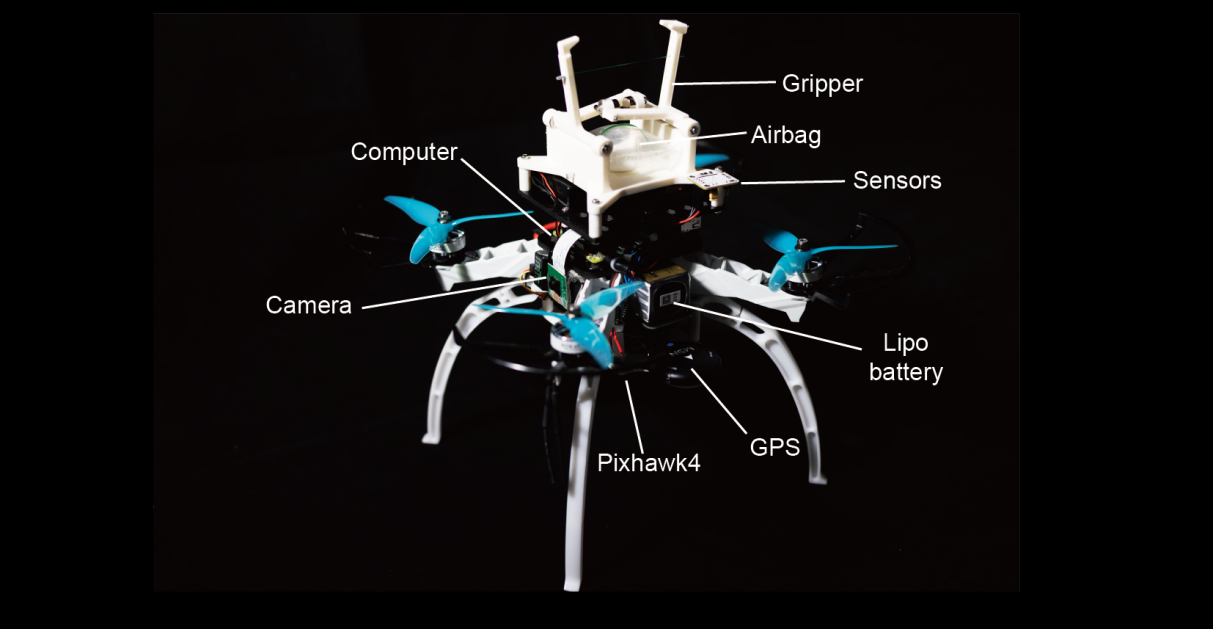


**Fig. S16.** The composition of our UAV equipped with MTRGs.


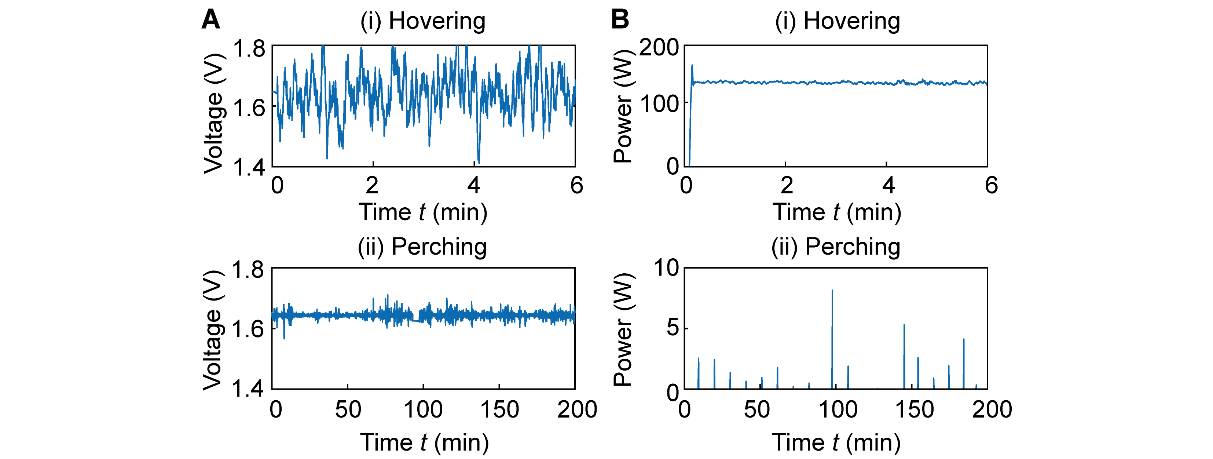


**Fig. S17.** Comparison between hovering and perching of UAVs, including (A) environmental noise and (B) energy consumption.


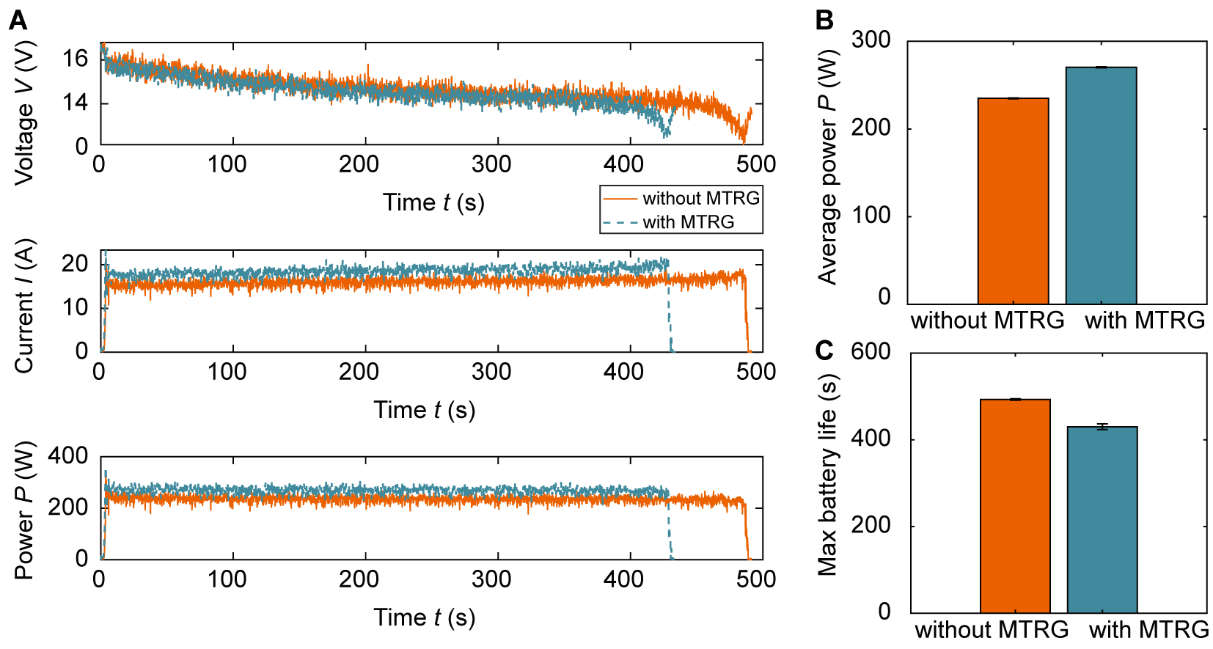


**Fig. S18.** The influence of MTRG on flight performance. (A) The variation of voltage, current, and power. Comparison of (B) average power and (C) maximum battery life.


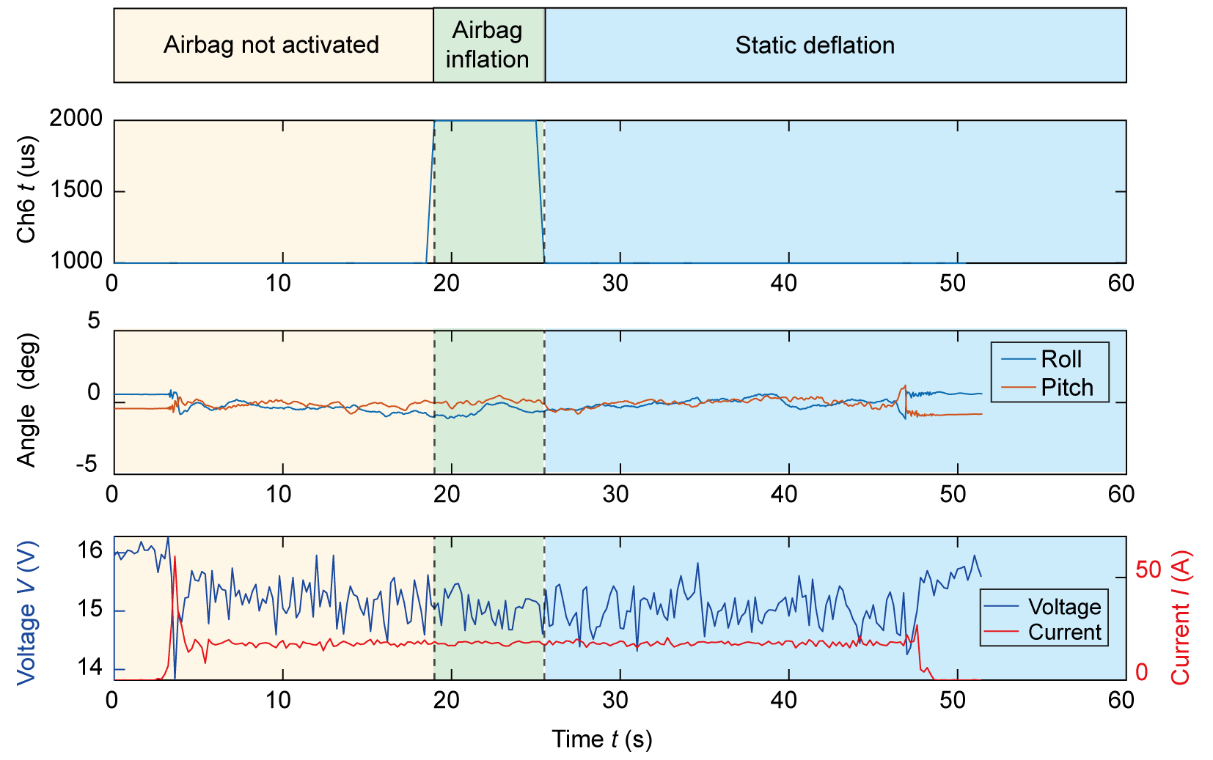


**Fig. S19.** Effects of both airbag inflation and static deflation on positioning accuracy.


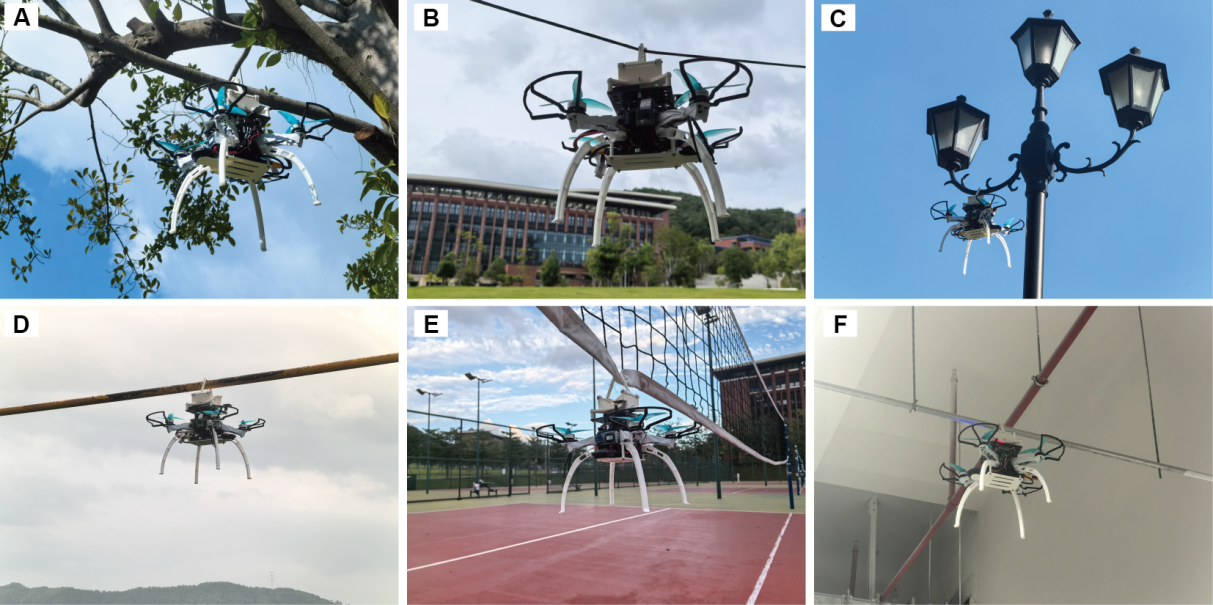


**Fig. S20.** UAV perching at different locations, including (A) tree branch, (B) electrical wire, (C) streetlight pole, (D) metal bar, (E) tennis net, and (F) indoor cable conduit.


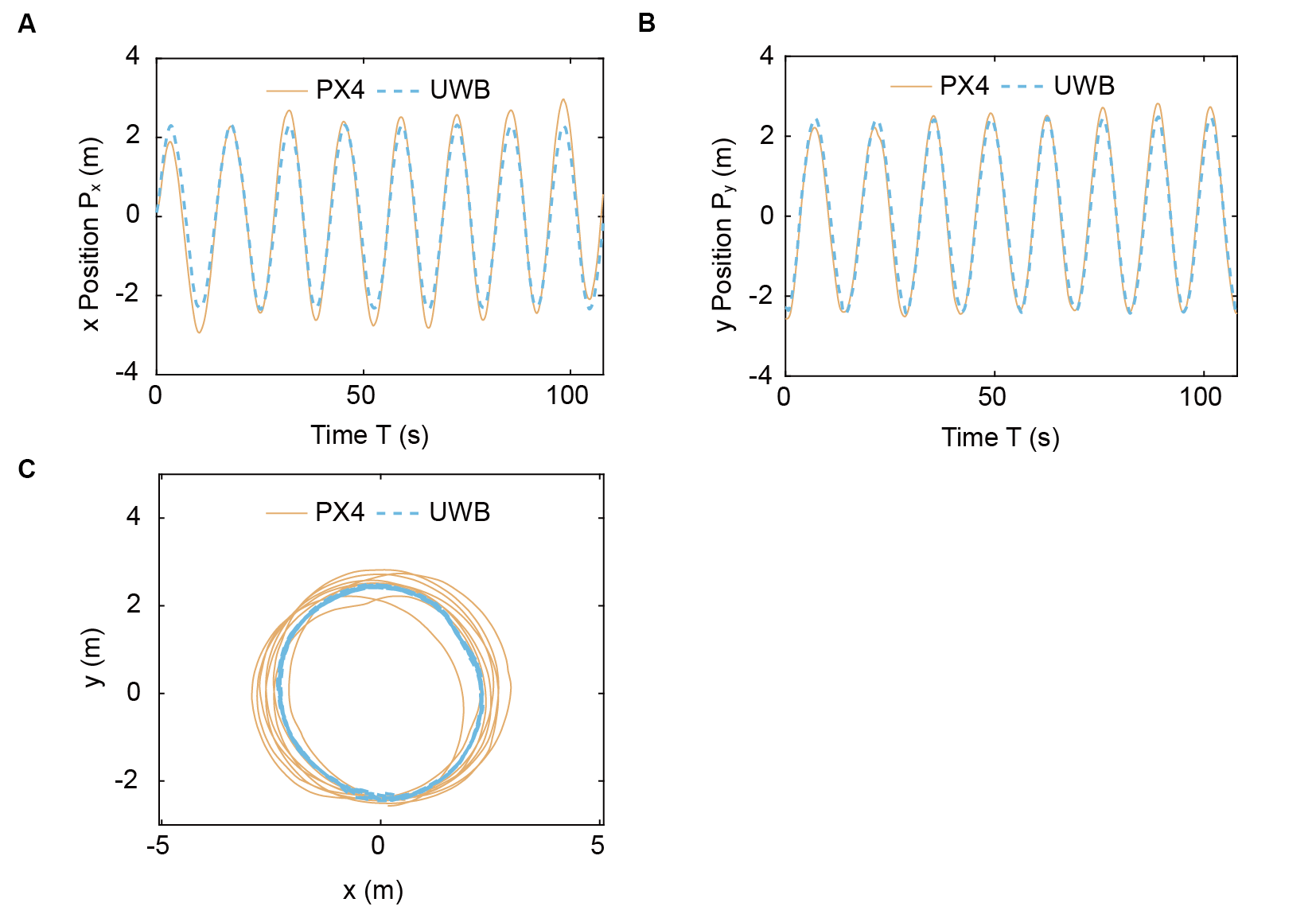


**Fig. S21.** Comparison of UAV GPS positioning and UWB system positioning, including (A) trajectory comparison, (B) *x*-direction and (C) *y*-direction positioning comparison.

**Table S1.** **Performance comparison between our robotic gripper and existing bistable robotic grippers**

| Reference | Mechanism type | Active actuator | Trigger force (N) | Failure force (N) | Failure/ trigger force ratio | Duration  (ms) | Mass  (g) | Payload/ weight ratio | Active energy barrier modulation |
| --- | --- | --- | --- | --- | --- | --- | --- | --- | --- |
| [25] | Bistable tensegrity | No | 1.00 | 0.50 | 0.50 | 186 | 56.33 | 3.10 | No |
| [36] | Soft bistability | No | 1.50 | 0.34 | 0.23 | 800 | 4.20 | 8.10 | No |
| [31] | Bistable origami | No | 1.57 | 2.80 | 1.78 | 1730 | 5.00 | 57.14 | No |
| [37] | Bistable origami | Yes | 1.50 | 10.00 | 6.70 | 80 | >1000 | <1.02 | No |
| [38] | Soft bistability | Yes | 40.00 | 23.75 | 0.59 | 110 | 536 | 4.47 | Yes |
| [39] | Bistable origami | Yes | 4.00 | 6.00 | 1.50 | 300 | - | - | Yes |
| [40] | Pneumatic bistability | Yes | 5.00 | 3.44 | 0.69 | 130 | - | - | Yes |
| [41] | Bistable origami | Yes | 8.50 | 24.88 | 2.93 | 300 | 35 | 72.54 | Yes |
| [16] | Bistable beam | Yes | 0.66 | 12.08 | 18.18 | 25 | 150 | 9.32 | Yes |
| [S2] | Tunable bistability | Yes | 0.12 | 1.72 | 14.33 | 200 | - | - | Yes |
| [S3] | Bistable + SMA | Yes | - | 15.96 | - | 300 | 50 | 32.57 | Yes |
| [S4] | Bistable + SMA | Yes | - | 1.96 | - | 71 | 20 | 10 | Yes |
| [S5] | Pneumatic bistability | Yes | - | 0.87 | - | 150 | 10 | 8.9 | Yes |
| **This work** | **Magnetic bistability** | **No** | **0.12** | **35.05** | **291.83** | **42** | **75.23** | **47.54** | **No** |

**Table S2. Performance comparison between our robotic gripper and other robotic grippers for perching**

| Reference | Total mass (g) | Gripper mass (g) | Trigger force (N) | Failure force (N) | Failure/ trigger force ratio | Payload/ weight ratio | Duration (ms) | Energy consumption | Application scenarios | Perching mode |
| --- | --- | --- | --- | --- | --- | --- | --- | --- | --- | --- |
| [16] | 548.1 | 132.21 | 0.66 | 12.08 | 18.30 | 9.32 | 25 | For energy barrier modulation | Diameters 5-25 mm | Hanging |
| [18] | 750 | 250 | - | - | - | - | 50 | For balance during perching | Diameters 38-165 mm | Bird-like grasping |
| [20] | 1800 | 300 | - | 80 | - | 27.21 | 65 | For actuation | Diameters 20-50 mm | Hanging |
| [26] | 593 | 28 | 0.42 | 36.26 | 86.33 | 132.14 | - | None | Diameter ~30 mm | Hanging |
| [27] | 1140 | 150 | 24 | 176 | 7.33 | 119.73 | 4 | For actuation | Diameters 55-115 mm | Bird-like grasping |
| [31] | 19.98 | 5 | 1.57 | 2.8 | 1.78 | 57.14 | 1730 | None | Diameters 8-38 mm | Hanging |
| [38] | 1586 | 536 | 40 | 23.47 | 0.59 | 4.47 | 110 | For active triggering | Diameters 80-140 mm | Bird-like grasping |
| [43] | 40 | 8 | 0.39 | 2.12 | 5.44 | 27.04 | - | For state transitions | Width 3.7-36.4 mm | Hanging |
| [S6] | 1734 | 402 | 0.8 | 16.23 | 20.2875 | 4.12 | - | For active triggering | Diameters 40-60 mm | Bird-like grasping |
| [S3] | 500 | 50 | - | 15.96 | - | 32.57 | 300 | For triggering and intermittent grip maintenance | Diameters 40-100 mm | Bird-like grasping |
| [S7] | 172 | 38.8 | 1.6 | 9.5 | 5.9375 | 24.98 | 100 | For triggering | Diameters 30-50 mm | Bird-like grasping |
| [S4] | 70.5 | 20 | - | 1.96 | - | 10 | 71 | For triggering | Diameters 20-70 mm | Bird-like grasping |
| [S8] | 2700 | - | - | ~60 | - | - | 100 | For triggering | Diameters 35-85 mm | Hanging |
| **This work** | **1033.82** | **75.23** | **0.12** | **35.05** | **293.38** | **47.54** | **42** | **None** | **Diameters ≤30 mm** | **Hanging** |

**Table S3. Parameter determination of bistable robotic gripper**

| Parameter | Value |
| --- | --- |
| *L*_AB_ | 4.52 mm |
| *L*_BQ_ | 51.66 mm |
| *L*_AA′_ | 66 mm |
| *L*_AD_ | 66 mm |
| Thickness of a single magnet $\text{l}$ | 14 mm |
| Triggering angle of the fingers *θ*ₜ | 5° |
| Closing angle of the fingers *θ*_c_ | 30° |

Video S1. Gripper assembly

**Video S2:** Dynamic disturbance-triggering experiments

**Video S3:** High-speed video of gripper triggering

**Video S4:** Gripper trigger experiment

**Video S5:** Gripper failure force test experiment

**Video S6:** Dynamic disturbance-failure force experiments

**Video S7:** Airbag recovery experiment

**Video S8:** UAV perching experiment

**Video S9:** Perching experiments in different scenarios

**Reference**

[S1] Robertson W, Cazzolato B, Zander A. A simplified force equation for coaxial cylindrical magnets and thin coils [J]. IEEE Transactions on magnetics, 2011, 47(8): 2045-2049.

[S2] X. Sheng, Z. Wei, W. E. I. Zhong, Y. Yang, S. U. N. Cong, and L. I. U. Zhen, “A rigid-flexible coupling multimodal gripper based on an adjustable bistable mechanism,” ROBOT, vol. 47, no. 1, pp. 22–31, Jan. 2025, doi: 10.13973/j.cnki.robot.240141.

[S3] A. Hammad, M. Süer, and S. F. Armanini, “A lightweight bioinspired SMA-based grasping mechanism for flapping wing MAVs,” Biomimetics, vol. 10, no. 6, p. 364, June 2025, doi: 10.3390/biomimetics10060364.

[S4] Y. Liu, Y. Mo, X. Liang, Y. Jiang, J. Li, and S. Wei, “Micro-UAV with ant-inspired bistable gripper for adaptive perching and wildlife detection,” in 2025 IEEE/RSJ International Conference on Intelligent Robots and Systems (IROS), Oct. 2025, pp. 10289–10294. doi: 10.1109/IROS60139.2025.11247399.

[S5] Z. Zhang et al., “Pneumatically actuated soft gripper with bistable structures,” Soft Rob., vol. 9, no. 1, pp. 57–71, Feb. 2022, doi: 10.1089/soro.2019.0195.

[S6] Y. Zhao et al., “Design and Validation of a Biomimetic Leg-Claw Mechanism Capable of Perching and Grasping for Multirotor Drones,” Biomimetics, vol. 10, no. 1, Art. no. 1, Dec. 2024, doi: 10.3390/biomimetics10010010.

[S7] K. C. V. Broers and S. F. Armanini, “Repeatable energy-efficient perching for flapping-wing robots using soft-grippers,” Bioinspiration Biomimetics, vol. 20, no. 6, p. 66017, Nov. 2025, doi: 10.1088/1748-3190/ae18a8.

[S8] Y. Li et al., “Design and Control of a Perching Drone Inspired by the Prey-Capturing Mechanism of Venus Flytrap,” Sept. 16, 2025, arXiv: arXiv:2509.13249. doi: 10.48550/arXiv.2509.13249.
